# Supplementary material for: Development and piloting of a modular evaluation tool for patient and public involvement in health services research: protocol of a mixed-methods study
Source: BMJ Open. 2025 Dec 2;15(12):e110547. doi: 10.1136/bmjopen-2025-110547 (PMC12706111; doi:10.1136/bmjopen-2025-110547)
Supplement: Author's manuscript [file bmjopen-2025-110547.draft_revisions.pdf]

BMJ Open is committed to open peer review. As part of this commitment we make the peer review history of every article we publish publicly available.

When an article is published we post the peer reviewers' comments and the authors' responses online. We also post the versions of the paper that were used during peer review. These are the versions that the peer review comments apply to.

The versions of the paper that follow are the versions that were submitted during the peer review process. They are not the versions of record or the final published versions. They should not be cited or distributed as the published version of this manuscript.

BMJ Open is an open access journal and the full, final, typeset and author-corrected version of record of the manuscript is available on our site with no access controls, subscription charges or pay-per-view fees (http://bmjopen.bmj.com).

If you have any questions on BMJ Open's open peer review process please email info.bmjopen@bmj.com

BMJ Open

Development and Piloting of a Modular Evaluation Tool for Patient and Public Involvement in Health Services Research - Protocol of a Mixed-Methods Study

Journal:	*BMJ Open*
Manuscript ID	bmjopen-2025-110547
Article Type:	Protocol
Date Submitted by the Author:	08-Sep-2025
Complete List of Authors:	Seeralan, Tharanya; University Medical Center Hamburg-Eppendorf, Department for Medical Psychology, Center for Psychosocial Medicine<br>Oster, Lena; University Medical Center Hamburg-Eppendorf, Department of Medical Psychology, Center for Psychosocial Medicine<br>Jones, Julia; University of Hertfordshire, Centre for Research in Public Health and Community Care, School of Health, Medicine and Life Sciences<br>Mathie, Elspeth ; University of Hertfordshire, Centre for Research in Public Health and Community Care, School of Health, Medicine and Life Sciences<br>Härter, Martin; University Medical Center Hamburg-Eppendorf, Department of Medical Psychology, Center for Psychosocial Medicine<br>Brütt, Anna Levke; University Medical Center Hamburg-Eppendorf, Department of Medical Psychology, Center for Psychosocial Medicine; Carl von Ossietzky University of Oldenburg School VI - Medicine and Health Sciences, Department of Health Services Research, Junior Research Group for Rehabilitation Sciences
Keywords:	Patient Participation, Health Services, Community-Based Participatory Research, QUALITATIVE RESEARCH, Surveys and Questionnaires

SCHOLARONE™ Manuscripts

Development and Piloting of a Modular Evaluation Tool for Patient and Public Involvement in Health Services Research - Protocol of a Mixed-Methods Study

Tharanya Seeralan[1], Lena Oster[1], Julia Jones[2], Elspeth Mathie[2], Martin Härter[1], Anna Levke Brütt*[1, 3]

[1]Department of Medical Psychology, Center for Psychosocial Medicine, University Medical Center Hamburg-Eppendorf, Hamburg, Germany

[2] Centre for Research in Public Health and Community Care, School of Health, Medicine and Life Sciences, University of Hertfordshire, United Kingdom

[3] Department of Health Services Research, Junior Research Group for Rehabilitation Sciences, School of Medicine and Health Sciences, University of Oldenburg, Oldenburg, Germany

***Correspondence to:**

PD Dr. phil. Anna Levke Brütt, Dipl.-Psych., M. Sc.

Department of Medical Psychology

University Medical Center Hamburg-Eppendorf

Martinistraße 52, Building W26, 20246 Hamburg

Email address: a.bruett@uke.de

For peer review only

ABSTRACT

Background: There is limited evidence regarding the outcomes and impacts of Patient and public involvement (PPI) in research, mainly based on narrative studies. Existing frameworks for supporting and evaluating PPI often require adaptation to specific contexts, and comprehensive instruments are needed. From an international perspective, strengthening the scientific foundation that underpins PPI is crucial to generate stronger evidence to understand which approaches work best, in which contexts, and with what effects.

Objectives: To promote PPI implementation in German health research, this project aims to 1) establish an evaluation framework 2) develop a modular evaluation tool in form of a questionnaire, and 3) pilot and psychometrically validate the tool.

Methods and Analysis: A three-phase mixed methods approach will be employed, integrating qualitative and quantitative data. First, we will explore with researchers, research partners, and other stakeholders in health services research, what contributes to meaningful and successful PPI through a web-based survey and focus groups. Findings are discussed in a codesign workshop in which participants agree on an evaluation framework based on a LOGIC model. Second, items from international instruments that evaluate PPI are deductively assigned to the evaluation framework. Further items are developed based on the focus groups from phase 1. Cognitive pretests and qualitative review will be conducted with researchers and patients in order to refine the item pool and develop the evaluation tool. Third, the evaluation tool with modules for researchers and patients will be piloted in a web-based survey. Data analysis will include thematic analysis for qualitative data and descriptive and psychometric analyses for quantitative data. A participatory research team will provide ongoing support throughout all project phases.

Ethics and Dissemination: Ethical approval has been obtained from the Local Ethics Committee. The study will follow the principles of the Helsinki Declaration and good scientific practice. Results will be disseminated at national and international conferences, public symposiums, and in peer-reviewed journals, contributing to the internationally developing field of PPI in research and addressing relevant research gaps.

Keywords: Patient Participation, Community-Based Participatory Research, Health Services, QUALITATIVE RESEARCH, Surveys and Questionnaires

For peer review only

Strengths and limitations of this study

- This study employs a mixed methods approach, integrating qualitative (e.g., focus groups, cognitive pretests) and quantitative (e.g., surveys, psychometric validation) methods.
- It involves multiple stakeholders, including researchers, research partners, and patients, to ensure comprehensive insights and participatory development of the evaluation framework and modular tool.
- It addresses the lack of comprehensive frameworks for evaluating PPI in German health research, providing essential tools to support, report, and systematically assess the impacts of PPI.

For peer review only

BACKGROUND

Through patient and public involvement (PPI) in research, the knowledge, skills and expertise of those directly affected by health issues and/or research outcomes can be considered. Especially in the United Kingdom, the United States, and Canada, it is widely accepted that involving patients and the public[1] in various roles (1) is possible at all stages of the research process (2). They can prioritize research questions, assist during study implementation, review and interpret study results, and contribute to the dissemination of findings (3). As a consequence, funders, policy-makers and research organizations increasingly expect patients to be involved, particularly in health services research.

There are specific national programs that support PPI by promoting and providing infrastructure. In the UK, support was initially provided by INVOLVE, a national advisory group funded by the UK government. INVOLVE has since been integrated into the National Institute for Health and Care Research (NIHR) and its functions are now part of broader NIHR initiatives. In the USA, The government-sponsored Patient-Centered Outcomes Research Institute (PCORI) strengthens patient and stakeholder relationships by building networks. Moreover, PCORI guides and supports PPI within the research it funds. In Canada, the Institutes for Health Research Strategy for Patient-Oriented Research (SPOR) acts as a funder and facilitator for PPI in research. Beneficial as well as challenging outcomes and impacts of PPI have been demonstrated. Systematic reviews (4) and umbrella reviews (5) suggest that researchers report a greater understanding and deeper insights into their research area as well as personal benefits such as motivation. Challenges include feeling uncomfortable sharing power over research and investing additional time in partnerships (4, 5). Patients, on the other hand, report benefits including increased knowledge and skills, a more positive attitude towards research, improved access to information, and personal benefits such as feeling empowered. However, patients also reported feelings of not being listened to or taken seriously by researchers, as well as feeling overwhelmed by tasks and responsibilities during the research process (4, 5). In terms of outcomes and impacts on the research process, PPI can lead to more patient-centered research questions. Moreover, the comprehensibility of patient information material, questionnaires and interview schedules may be improved. PPI also accounted for more appropriate recruitment strategies and enhanced implementation and dissemination (6, 7). Additionally, PPI may improve the health-related outcomes of the involved patients (8). Challenges regarding the organization of research projects reflect the additional time required and the insufficient financing of PPI (7, 9). Most of the reported outcomes and impacts originate from narrative studies. As a consequence, the evidence-base in PPI concerning the outcomes, impact and good practice criteria is rather limited (4, 5, 7, 9, 10).

There are several reasons why adequate evidence has not been generated thus far:

First, some researchers do not see a need to generate evidence on the outcomes and impacts of PPI, as they see it as a democratic right with intrinsic value (10). The international debate on the need to

For peer review only

measure the outcomes and impact of PPI is ongoing (11). For example, Staley and Barron emphasize that PPI supports mutual learning between patients and researchers and does not require measuring its impact (12). In contrast, Staniszewska points out that measuring outcomes and impacts strengthens the evidence base of PPI (13). Boivin et al. further emphasized that meaningful involvement requires sound evaluation (14). However, a compromise is that PPI evaluation should not be limited to measuring outcomes and impacts, but should also include the process and context (10). Second, there is a heterogeneous terminology (5), and a poor reporting of PPI (7, 8, 15, 16), limiting the detection of its outcomes and impacts. Recently, consensus-informed guidelines for reporting PPI in research (Guidance for Reporting Involvement of Patients and the Public; GRIPP2) have been published on the EQUATOR webpage (www.equator-network.org). The GRIPP2 includes minimum reporting requirements in a short form (17). In addition to defining the aims related to PPI and the methods used, articles and reports should also include impacts and outcomes - positive as well as negative - along with their measurement. While challenges such as tokenistic PPI may still exist (18), this improves the quality, transparency, and consistency of PPI reporting (19). Third, there are numerous frameworks for supporting PPI in research (11). A comprehensive review and synthesis of PPI frameworks identified five different types: power-focused, priority-setting, study-focused, report-focused and partnership-focused frameworks. It also revealed that most published frameworks are rarely used outside the groups that developed them. Therefore, the authors concluded that these frameworks are difficult to transfer to other contexts and require adaptation for specific settings (11). Fourth, available tools for evaluating PPI do not take into account its complexity, as both the process and the context need to be described. Furthermore, outcomes and impacts on patients, researchers and the research itself need to be considered (20).

There is an increasing number of tools available for evaluating PPI (21). For example, the Public Involvement Impact Assessment Framework (PiiAF) aims to help researchers plan and assess the impacts of PPI. It compiled a set of questions on the values, approaches, focus, study design and impacts. PiiAF can assist in documenting the quality of PPI in research projects (22). It can be tailored to specific projects, but there are no defined items. PCORI uses the Ways of Engaging-Engagement Activity Tool (WE-ENACT) to collect information about PPI from researchers (23) and patients (24). Stocks et al. (25) developed an instrument to evaluate PPI from the patient's perspective focusing on the quality of involvement (26). The instrument accounts for both personal (e.g., feeling valued, achieving goals, feeling empowered) as well as contextual factors (e.g., research participation, previous experience). Outcomes and impact on the research process are only implied (26). The Public Patient Engagement Evaluation Tool [PPEET; (27)] evaluates its quality, including four principles: integrity of design and process, influence and impact, participatory culture, collaboration and common purpose. Different versions for researchers and patients are available. Another Canadian instrument, the Patient Engagement In Research Scale (PEIRS), is one of the very few instruments that underwent psychometric evaluation (28, 29). The items reflect procedural requirements, convenience, contributions, team

For peer review only - http://bmjopen.bmj.com/site/about/guidelines.xhtml

environment and interaction, support, feelings of being valued and benefits. While the PiiAF lacks standardization, the WE-ENACT tools do not account for the context. Similarly, the instrument by Stocks et al. (25) and the PPEET(27) fail to include information on how PPI is conducted. The PEIRS (28, 29) does not encompass the perspective of researchers.

To date, none of these tools provides a comprehensive evaluation of PPI with regard to the context, process, outcomes or impacts from different perspectives. Moreover, their scientific rigor in the development and validation process requires improvement (21). In summary, from an international perspective, there is a need to strengthen the science behind PPI to generate stronger evidence to understand which PPI approaches work best, in which contexts and with what effects (16, 21, 30-35).In addition to these general methodological aspects, country-specific contexts must also be considered. The implementation of PPI is heterogeneous. For example, in Germany PPI has not yet been systematically implemented in health services research. Among the 683 studies (published up to 2009) included in the extensive review on PPI conducted by Boote et al. (36) only two originated from Germany. This has not changed over the past decade. In 2021, Biddle et al. concluded that the implementation of PPI is very heterogeneous and not yet firmly established or formalized in European health services research. They refer to a lack of infrastructure, guidance and support as potential barriers (37).

Recently, German research funders have introduced PPI into health research as a requirement within project proposals, aiming to drive its practice. Meanwhile, some documents and principles have been published that provide orientation for participatory health research in Germany (38-42). However, despite these important contributions, a comprehensive and systematic framework for evaluating PPI in health service research in Germany remains absent. The lack of an overarching evaluation concept may risk tokenistic approaches to PPI, as we have learned from international research (19, 43, 44). Therefore, an evaluation framework for PPI is needed to adequately support, report and evaluate PPI (11) in health services research in Germany. Furthermore, while realizing PPI has been central in various of our previous studies (45-50), we were also confronted with the lack of adequate instruments to evaluate the process, perceived impact, and potential harms of PPI. Thus far, there is a limited understanding of what constitutes meaningful and successful PPI from the perspectives of both researchers and patients in health services research in Germany.

OBJECTIVES

Therefore, the study objectives are (1) to establish a theoretical evaluation framework, (2) based on this, to develop a modular evaluation tool as a practical questionnaire for various stakeholders and (3) to pilot the modular evaluation tool including psychometric testing.

For peer review only - http://bmjopen.bmj.com/site/about/guidelines.xhtml

We would like to prepare an evaluation framework for PPI in health services research in Germany, considering the context, process, outcomes and impacts. The objective of establishing an evaluation framework is related to the following research questions:

1. *What does meaningful and successful involvement in health services research look like for researchers and patients in Germany?*
2. *How should the evaluation of PPI be approached in health services research in Germany?*

We will acknowledge the international literature and will explore the specifics of PPI in health services research in Germany. Items will be devised acknowledging existing instruments as well as qualitative survey and interview data. Related research questions are:

3. *Do the devised items address the consented evaluation framework?*
4. *Are the devised items of the modular evaluation tool comprehensive and relevant?*

The developed modular evaluation tool will undergo validity and reliability testing.

5. *Does the modular evaluation tool produce stable and consistent results?*

We will place further emphasis on PPI in the evaluation design and reporting by involving a participatory research team in all phases of the research process.

METHODS AND ANALYSIS

Study design

The proposed 3-year mixed-methods project comprises three phases. Each phase examines one of the objectives described above (figure 1).

– please insert figure 1 about here –

Study population

The study will focus on the perspectives of researchers, patients, and stakeholders with PPI experience within the context of German health services research. Adult patients (≥ 18 years) with lived experience in treatment-relevant health conditions and experience with the healthcare system will be included. Patients with limited proficiency in the German language, as well as those with severe impairments (e.g., cognitive impairment or lower quality of life due to illness), will be excluded. Researchers and patients will be recruited through databases on publicly funded health services research and via recently established PPI networks (e.g., DNVF patient advisory board, personal contacts). Detailed information regarding specific study population and recruitment strategy will be provided aligning with the description of the three phases and work packages presented below.

Relevance of sex, gender and/or diversity

Special attention will be given to considering the diverse perspectives of researchers and patients. In web-based surveys, researchers and patients with relevant experiential knowledge about patient involvement can contribute. The community of health services researchers in Germany is heterogeneous (51), but diversity has not been investigated thus far. Patients involved in this research are often white, female and retired (52). It is therefore likely that, in aiming to engage individuals and community groups already experienced in participatory research, participating patients may have a narrow set of sociodemographic characteristics, and the views of a more diverse population may be underrepresented. We will include diversity aspects in questions regarding sociodemographic characteristics and will therefore be able to at least describe them in the survey sample. Setting up a web-based survey will not produce selection bias among researchers. However, patients who have previously been involved in research will presumably be familiar with the technique.

For the qualitative study parts, we will stratify our sampling strategies based on relevant sociodemographic aspects to reflect diversity. We will sample researchers with regard to gender, age/research stage, and the field of expertise/research methods. With regard to patients, we consider gender, age and education to be relevant. In addition, we will include patients with different health conditions and associated networks (individual patient vs. member/representative in a patient organization). By asking for sociodemographic characteristics, we will be able to report on diversity in the qualitative study parts.

Moreover, we aim to identify a diverse group of people, by gender, age and educational background, to join the PPI group that we are calling the participatory research team. This team should also include experience with different kinds of diseases and networks as well as experience with being a patient partner. On the one hand, we assume that patient partners acquire knowledge that is useful for future participatory research teams (53, 54). On the other hand, we will recruit additional persons without prior experience in PPI. We assume that skills such as confidence in articulating oneself in a formal meeting would support an interest in joining a participatory research team. As suggested by Thompson et al. (53) and Lander et al. (55), we will reflect on which input will be most useful, provide reasons for why certain participants were selected and discuss how potential recruiting and representativeness limitations impact the process and results.

For peer review only

Phase 1: Establishing an evaluation framework

Aim and methods

We aim to contribute to a scientifically sound, comprehensive evaluation framework for PPI in health services research in Germany based on a logic model (56). It will be used to describe the causal processes through which PPI produces outcomes and impacts. Information on meaningful and successful PPI in health services research from the perspective of researchers and patients will be gathered by

conducting a web-based survey and focus group interviews. Results will be presented in a co-design workshop to establish an evaluation framework (see figure 1).

a. Web-based survey

The web-based survey consists of different modules for researchers and patients. They will be recruited through several time-shifted strategies: a) identification and invitation of principal investigators of publicly funded health services research, b) identification and invitation of researchers and patients (or their representatives) involved in publicly funded health services research that implements PPI, and c) through personal knowledge (e.g., Deutsches Netzwerk Versorgungsforschung (DNVF), including its patient advisory board).

Recruitment and procedures

Principal investigators of publicly funded projects in the field of health services research (German Research Foundation (Deutsche Forschungsgemeinschaft, DFG), Federal Ministry of Research, Technology and Space (Bundesministerium für Forschung, Technologie und Raumfahrt, BMFTR), Federal Ministry of Health (Bundesministerium für Gesundheit, BMG), innovation fund of The Federal Joint Committee (Gemeinsamer Bundesausschuss, G-BA) can be identified using databases (e.g., funded projects information system (GEPRIS), funding catalogue of the BMFTR). The inclusion criteria specify that the project must be conducted in Germany, initiated in 2010 or later, and aimed to gain knowledge about healthcare. In the GEPRIS Database currently lists 78 researchers, while the BMBF funding catalogue contains 571 projects. Researchers will be contacted via email and invited to participate in a linked web-based survey. With regard to sampling strategy 2, these principal investigators can name further researchers and patients for participation in the survey. Four weeks after the second reminder email has been sent to the principal investigators, sampling strategies b) and c) will be applied. Links to the web-based survey will be sent via email. Two reminders will be sent (57). The web application Research electronic data capture (REDCap) will be used to implement the web-based survey (58). Informed consent will be obtained online.

In module B, all participating principal investigators and researchers and patients will provide information on individual characteristics. Researchers will answer questions on age, gender, seniority (19) and patients will be asked questions with regard to age, gender, health condition with lived experience and membership of self-help organization. In module C, the participants are presented with information on PPI and will be asked to rate different elements of the frameworks for supporting PPI in research according to their relevance (11, 38-41). For both aspects, a nine-point Likert scale (1=not relevant; 9=very relevant) will be used. In addition, open-ended questions will be asked about whether any relevant aspects are missing. At the end of the web-based survey, participants can consent to be contacted for further interviews or surveys in the project, which requires storing their email address

For peer review only - http://bmjopen.bmj.com/site/about/guidelines.xhtml

and further details (such as project and investigator characteristics) in a project database and/or for receiving information about the project results.

Data analysis

To analyse the project and participant characteristics, we will calculate the median, mean and standard deviation (SD) for the interval data and frequencies for the nominal items. In the analysis of the ratings (module C), the median, mean and SD will be calculated. All data will be analysed using IBM SPSS Statistics 29 (59). The descriptive analysis of the web survey is intended to provide an overview of the relevant domains of internationally developed PPI frameworks from the perspective of health services researchers in Germany.

b. Focus groups

Focus groups (60) will be conducted to validate and expand the findings of the web-based survey. Researchers as well as patients experienced in PPI will be recruited. An interview guide will be developed based on the different elements of the logic framework and will be adapted for researchers and patients. The conduction of focus groups with patients will be discussed and planned together with the participatory research team.

Recruitment and procedures

Six focus groups will take place, separately with researchers and patients, with 5 to 7 participants moderated by two members of the project team. Empirical studies have shown that 3 to 6 focus groups will be enough to discover 90% of the themes (61). We will recruit participants as outlined. We aim to include a broad range of researchers and patients experienced with PPI in health services research. We will aim for maximum variation in the researcher sample with regard to early-stage and advanced researchers, different fields of expertise (e.g., mental health services research) and diversity with regard to individual participants (e.g., gender). The patient sample is planned to include diversity with regard to health conditions and associated networks (e.g., individual patients vs. member/representative in/of patient organization) as well as sociodemographic factors (e.g., gender, age, educational background). The interview guide will be tailored to the participant groups. The core questions concern the context, process, outcomes and impact of PPI. A detailed interview guide will be developed involving the participatory research team.

Data analysis

The focus group interviews will be audio-recorded and transcribed verbatim by a professional transcription company. Afterwards, qualitative thematic analysis (62) will be applied. Relevant passages will be coded using a deductive category system derived from the interview guide. Furthermore, inductive coding to refine the main categories will be applied. There will be two coders, and intercoder

For peer review only

reliability will be assessed before the consensus discussions take place within the project team. For the documentation and analysis of the data, the software MAXQDA 2020 (63) will be used.

c. Codesign workshop

The codesign workshop is planned according to the suggestions of Greenhalgh and colleagues (11). It will be carried out with approximately 16 researchers, patients and further relevant stakeholders (e.g., representatives of the funding bodies or health insurances) in the field of health services research in Germany.

Recruitment, procedures and data analysis

Researchers and patients who consented to participate in the previous study parts will be considered. At least four persons from each group should participate in the codesign workshop. Criteria with regard to diversity, only gender and age, will be applied due to the low number of participants in the codesign workshop. Stakeholders should reflect the field of health services research, e.g., representatives of the funding bodies or health insurance. The codesign workshop will be a whole day workshop (6 hours). It will start in plenary to present the condensed results of the web-based survey and the themes identified in the focus groups. Participants will have the opportunity to ask questions and to discuss the themes. Following the suggestions (11), each participant will be asked to choose up to 10 themes that they think are the most important as indicated by multichoice voting. Participants can indicate their preferences by marking points at the respective themes, which are displayed on flip charts. Accordingly, votes are collected from all participants and will be available in real-time. This information will be available for the following group work aiming at relating the identified themes to the logic model. Participants will work in four groups of approximately four people. Each group should include a mix of researchers, patients and further stakeholders. Each group will be assigned to one of the domains of the logic model (context, process, outcome and impact). Participants can use the presented themes and choose those that reflect the respective domain of the logic model. Afterwards, the preliminary version of the evaluation framework will be presented and discussed in plenary. In particular, themes that have been chosen by more than one group will be discussed and specified. Then, participants will return to the groups to refine their domain based on the discussions. Again, the group will present their domain. The process is repeated if participants bring up further discussion points. It is possible that the evaluation framework will include different domains for researchers and patients. Finally, the framework as a whole will be discussed in plenary and accepted in consensus.

Phase 2: Development of a modular evaluation tool

Aim and methods

For peer review only - http://bmjopen.bmj.com/site/about/guidelines.xhtml

The development of the modular evaluation tool in the form of a questionnaire is based on Streiner et al.'s (64) recommendations, including devising and reviewing items. Aspects of validity will also be considered (65).

a. Item development

In the first step, we generate an item pool based on the existing instruments. In the second step, we will use the themes developed within the analysis of the free-text answers in the web-based survey and focus groups to develop additional items. As numerous instruments have already been developed internationally, as displayed in the review by Boivin et al. (21), we will acknowledge these by linking their items to the developed framework.

Procedures and data analysis

Since PPI is a dynamic field and several instruments have not been included (28, 29) in a previous systematic review (21), we will first update the review alongside the data collection during the first phase of the project (see figure 1). Afterwards, the items of all identified instruments will be systematically collected. Then, qualitative thematic analysis (62) will be applied using a deductive category system derived from the components of the consented evaluation framework (see phase 1). Each item will be linked to the content areas. The results will be displayed in a matrix (64). There will be two coders, and intercoder reliability will be assessed before consensus discussions will follow. For the documentation and analysis of the data, the computer program MAXQDA 2020 (63) will be used. Second, items from existing questionnaires for PPI evaluation that match the evaluation framework will undergo a standardized translation procedure, including forwards and backwards translations (66). Additionally, the research team will formulate additional items based on the themes derived from the free-text answers of the web-based survey and the focus groups. Ideally, the phrasing of the items is close to the original the statements of the participants. This process will result in a comprehensive item pool and the next step includes eliminating redundant or poorly worded items. An iterative process including the participatory research team will be used to refine the items. Afterwards, scaling will be specified. Finally, a preliminary version that undergoes cognitive pretesting will be defined. With regard to the proposed modular character of the evaluation tool, separate modules can be developed for researchers and patients.

b. Cognitive pretests

Procedures and data analysis

Cognitive pretests will use the 'Think Aloud' technique (67). We will invite 5 researchers and 5 patients who have already participated in the previous study phases to the first round. The researchers and patients will be asked to verbalize their thoughts that lead them to the answer while filling out their questionnaires. We plan to conduct cognitive pretests in person. Therefore, we will primarily recruit patients locally. The 'Think Aloud' will be recorded in writing and evaluated. Using the data from the

For peer review only - http://bmjopen.bmj.com/site/about/guidelines.xhtml

cognitive pretests, the researchers and the participatory research team will discuss and decide upon revisions. The decisions will be reached by consensus. The revised items will be presented in a second round of cognitive pretests involving another 5 researchers and 5 patients. After a further revision, the pretested and revised modular evaluation tool will undergo qualitative review.

c. Qualitative review

Procedures and data analysis

An online qualitative review will be conducted to investigate the face validity of the developed items. Researchers, patients and stakeholders who participated in the codesign workshop will be invited for the qualitative review. A web-based survey including all items will be administered using REDCap (58). Participants will judge each item with regard to its relevance on a 4-point scale (1: not relevant, 2: somewhat relevant, 3: quite relevant, 4: highly relevant). They will also be asked whether any important questions are missing. To ensure that all items are relevant and reflect the evaluation framework, the Content Validity Index (CVI) will be calculated (68). The CVI is computed as the number of experts giving a rating of "very relevant" for each item divided by the total number of experts. Values range from 0 to 1 where CVI > 0.79, the item is relevant; between 0.70 and 0.79, the item needs revisions; and if the value is below 0.70, the item is eliminated. Nevertheless, there should be at least three items per domain. Based on the scores, a revision will be made.

Phase 3: Piloting a modular evaluation tool

Aim and methods

The pilot study is intended to provide information on the reliability and validity of the developed modular evaluation tool. We will consider the relevant literature on the quality criteria for health outcome measurements (65, 69). With regard to reliability, we will focus on internal consistency and retest reliability, as there are no measurement standards for PPI evaluation tools. Additionally, we will account for the additional aspects of construct validity: factorial validity and convergent validity.

a. Web-based pilot study

Recruitment and procedures

Researchers and patients will be recruited by updating identifying the principal investigators of ongoing publicly funded health services research. Additionally, snowball sampling and sampling through personal knowledge will be applied. Researchers and patients will be invited to take part in web-based pilot testing of the modular evaluation tool. They will receive emails and individualised links. Reminders will be sent to improve the response rates (57). Participants are eligible when they are involved in a currently ongoing health research project that implements PPI. At the end of each survey, participants can also generate a code for specific ongoing projects and send links to the web-based pilot study to identify other project members. The code can be used to merge evaluations on specific projects.

For peer review only - http://bmjopen.bmj.com/site/about/guidelines.xhtml

Moreover, participants can choose whether they would like to receive a score sheet after closure of the pilot study.

Overall, the pilot study is intended to collect a total of at least 200 responses. Modules that can be answered by researchers or patients only should be answered by at least 100 participants. This will constitute an appropriate sample size providing insights into psychometric testing (70). A randomly chosen subsample of n=50 will be invited to fill in the core modules a second time after approximately 1 week. REDCap (58) will be used to implement the web-based survey. Informed consent will be obtained online.

The survey will include the items from the developed modular evaluation tool items as well as the PPEET (27) to establish convergent validity. The PPEET has previously been used for validation studies (29), and an authorized German version of the PPEET exists. Participating researchers and patients will also be asked to provide information on individual characteristics such as age and gender, as well as seniority for researchers and patients' health condition with lived experience for patients, respectively.

Data analysis

For sample characteristics, we will consider descriptive data. For item analysis, we will use descriptive data (N, mean, standard deviation, range, skewness, kurtosis and item difficulty). Internal consistency will be evaluated by Cronbach's α or the Kuder Richardson coefficient for dichotomous items. To assess test-retest reliability and construct validity, we will calculate correlations. We assume moderate correlations of the modules with related items of the PPEET. With regard to factorial validity, we will perform exploratory factor analysis. Further relevant aspects for reliability and validity testing, e.g., additional scales to be used and strengths of correlation, will be determined when the evaluation framework is agreed upon. All data will be analysed using SPSS (59). The aim is to obtain a modular tool with internally consistent scales (Cronbach's $\alpha > 0.70$) that captures a broad spectrum of factors relevant for PPI as identified in the evaluation framework.

Patient and public involvement

This researcher-initiated study was discussed with participatory research teams and patient advisory boards of previous and ongoing studies. For the proposed three-year project, a participatory research team comprising eight patient partners will be set up. The first meetings will be used to offer a research training to all patient partners. During the different phases of the project, they will for example, give feedback on study information and consent forms for the web-based survey, the patient focus groups and the codesign workshop. They will assist in recruiting strategies, discussing qualitative analysis, evaluating cognitive pretests and discussing the proposed findings of this study's dissemination of project progress and results. Further involvement can be agreed on during the research process. They will assist in writing papers by reviewing early drafts and providing feedback. We plan to involve the

For peer review only

participatory research team in all phases of the project through in-person and virtual meetings. Additional feedback from the participatory research team can be obtained via email between the meetings for specific aspects. Members of the participatory research team will be offered an expense allowance to acknowledge their contributions. The participatory process will be continuously evaluated through short surveys after each meeting and systematic reflections on process aspects e.g., team interactions, changes and impacts (26, 27).

ETHICS AND DISSEMINATION

Ethical considerations

Researchers and patients will participate in the study. This study does not involve any medical or psychological intervention that poses risk or harm to participants. The study will be conducted in accordance with the principles of the Helsinki Declaration and the standards of good scientific practice. All participants will be informed about the meaning, purpose and procedure of the study as well as the handling of the collected data. Informed consent will be obtained from all participants prior to taking part in the study. Approval from the local ethics committee and data security officer has been be obtained (LPEK-0889).

Data handling

New data will be generated in the empirical parts of the proposed project (survey, interviews). Researchers and patients will provide quantitative (e.g., ratings) as well as qualitative data (e.g., text, speech) in the web-based surveys, focus groups and co-design workshops. The web-based surveys conducted in phase 1 and phase 3, along with the qualitative review, will use the REDCap. REDCap supports data collection in accordance with the law (i.e., DGSVO), protecting the privacy of respondents (e.g., secure infrastructure, no cookies, no IP addresses in log files). For transcription of the focus groups, audio recordings will be uploaded to a secure and encrypted network and handled, ensuring data protection regulations. Quality checks of the transcripts will be performed by the interviewers and, if agreed on, by the interviewees. Audio recordings will be deleted after transcription, and the transcripts will be anonymised. Protocols will be made available for those who took park in the workshops or cognitive debriefings. This allows a quality check.

Informed consent for participation in the online surveys, focus groups, co-design workshops, and cognitive debriefing, and qualitative review will be obtained. The project team will store all data on a secure and encrypted network drive, with access to project team members. Declarations of consent and transcripts or protocols will be archived separately for 10 years before deletion. When compiling the results, no information will be published that could allow conclusions to be drawn about individual persons.

For peer review only

Dissemination plan

Attention will be given to effectively disseminating project results to researchers and patients. In accordance with open science practices, study deliverables, especially the evaluation framework and the evaluation tool, will be licensed under a creative commons licence and made available on the project website. For both deliverables, patient-friendly accompanying material developed with the participatory research team will be available. After piloting of the modular evaluation tool, it is aimed for providing an online tool that allows researchers and patients to evaluate PPI in specific projects. This holds the opportunity to monitor of PPI in health services research in Germany, but also to enable researchers and patients to receive individual feedback. The implementation of the online evaluation tool will be envisaged for a second funding period.

The study results will be presented to the participating researchers and patients in a public symposium. disseminated at national and international conferences and published in peer-reviewed journals. Moreover, there will be open access to the scientific results (e.g., open access articles), and we will provide lay-friendly summaries of published scientific articles on the project website. As is customary, the results will be reported back to the funder upon project completion.

Status of the study

The study started on January 1, 2025, data collection is planned to start on September 22, 2025 and the project is scheduled for completion on December 31, 2027 according to the current timeline. To date, approval from the local ethics committee has been obtained, we have started contacting interested patient partners for the participatory research team and initiated collaborations with international colleagues and cooperation partners. No substantive work within the different study phases has begun prior to the publication of this study protocol.

For peer review only

Statements

Declaration of Interest

The authors declare that they have no conflict of interest.

Tharanya Seeralan ORCID: 0000-0003-4431-0310
Lena Oster ORCID: 0009-0006-4744-2393
Elspeth Mathie ORCID: 0000-0002-5871-436X
Julia Jones ORCID: 0000-0003-3221-7362
Martin Härter ORCID: 0000-0001-74443-9890
Anna Levke Brütt ORCID: 0000-0002-4197-6048

Funding

This study was funded by The German Research Foundation (in German: Deutsche Forschungsgemeinschaft, DFG) under grant number 511611780. The sponsor had no influence on study design, data collection, management and analysis as well as interpretation of data, writing or publication process.

Author Contributions

ALB is the principal investigator who designed the study, wrote the grant proposal, and obtained the funding. ALB, TS, LO, JJ, EM and MH further refined the study by conceptualizing the research design, working out study processes, and coordinating the engagement of interested patient partners and associations. ALB and TS created the first draft of the manuscript. All authors contributed to critical revision of the manuscript for important intellectual content and approved the final version of the manuscript for publication.

Transparency Declaration

The corresponding author and manuscript guarantor [ALB] affirms that the manuscript is an honest, accurate, and transparent account of the study being reported, that no important aspects of the study have been omitted and that any discrepancies from the study registration will be reported.

Data Availability

The data that support the findings of this study will be available on reasonable request from the corresponding author [ALB].

For peer review only

Analytic Code Availability

The analytic code associated with this manuscript will be available on reasonable request from the corresponding author [ALB], only for purposes of reproducing results or replicating procedures.

Research Material Availability

The research materials associated with this manuscript will be available on reasonable request from the corresponding author [ALB], only for purposes of reproducing results or replicating procedures.

REFERENCES

1. Bird M, Ouellette C, Whitmore C, Li L, Nair K, McGillion MH, et al. Preparing for patient partnership: A scoping review of patient partner engagement and evaluation in research. Health expectations : an international journal of public participation in health care and health policy. 2020;23(3):523-39.
2. Forsythe LP, Ellis LE, Edmundson L, Sabharwal R, Rein A, Konopka K, et al. Patient and stakeholder engagement in the PCORI pilot projects: description and lessons learned. Journal of general internal medicine. 2016;31:13-21.
3. Forsythe L, Heckert A, Margolis MK, Schrandt S, Frank L. Methods and impact of engagement in research, from theory to practice and back again: early findings from the Patient-Centered Outcomes Research Institute. Quality of Life Research. 2018;27(1):17-31.
4. Brett J, Staniszewska S, Mockford C, Herron-Marx S, Hughes J, Tysall C, et al. A systematic review of the impact of patient and public involvement on service users, researchers and communities. Patient. 2014;7(4):387-95.
5. Hoekstra F, Mrklas KJ, Khan M, McKay RC, Vis-Dunbar M, Sibley KM, et al. A review of reviews on principles, strategies, outcomes and impacts of research partnerships approaches: a first step in synthesising the research partnership literature. Health Res Policy Syst. 2020;18(1):51.
6. McCarron TL, Clement F, Rasiah J, Moran C, Moffat K, Gonzalez A, et al. Patients as partners in health research: A scoping review. Health expectations : an international journal of public participation in health care and health policy. 2021;24(4):1378-90.
7. Brett J, Staniszewska S, Mockford C, Herron-Marx S, Hughes J, Tysall C, et al. Mapping the impact of patient and public involvement on health and social care research: a systematic review. Health Expect. 2014;17(5):637-50.
8. Halvorsrud K, Kucharska J, Adlington K, Rudell K, Brown Hajdukova E, Nazroo J, et al. Identifying evidence of effectiveness in the co-creation of research: a systematic review and meta-analysis of the international healthcare literature. J Public Health (Oxf). 2021;43(1):197-208.
9. Snape D, Kirkham J, Britten N, Froggatt K, Gradinger F, Lobban F, et al. Exploring perceived barriers, drivers, impacts and the need for evaluation of public involvement in health and social care research: a modified Delphi study. BMJ open. 2014;4(6):e004943.
10. Russell J, Fudge N, Greenhalgh T. The impact of public involvement in health research: what are we measuring? Why are we measuring it? Should we stop measuring it? Res Involv Engagem. 2020;6:63.
11. Greenhalgh T, Hinton L, Finlay T, Macfarlane A, Fahy N, Clyde B, et al. Frameworks for supporting patient and public involvement in research: Systematic review and co-design pilot. Health expectations : an international journal of public participation in health care and health policy. 2019;22(4):785-801.
12. Staley K, Barron D. Learning as an outcome of involvement in research: what are the implications for practice, reporting and evaluation? Research Involvement and Engagement. 2019;5(1):14.

For peer review only - http://bmjopen.bmj.com/site/about/guidelines.xhtml

13. Staniszewska S, Herron-Marx S, Mockford C. Measuring the impact of patient and public involvement: the need for an evidence base. Int J Qual Health Care. 2008;20(6):373-4.
14. Boivin A, Richards T, Forsythe L, Grégoire A, L'Espérance A, Abelson J, et al. Evaluating patient and public involvement in research. Bmj. 2018;363:k5147.
15. Mathie E, Wilson P, Poland F, McNeilly E, Howe A, Staniszewska S, et al. Consumer involvement in health research: A UK scoping and survey. Int J Consum Stud. 2014;38(1):35-44.
16. Shippee ND, Domecq Garces JP, Prutsky Lopez GJ, Wang Z, Elraiyah TA, Nabhan M, et al. Patient and service user engagement in research: a systematic review and synthesized framework. Health expectations : an international journal of public participation in health care and health policy. 2015;18(5):1151-66.
17. Staniszewska S, Brett J, Simera I, Seers K, Mockford C, Goodlad S, et al. GRIPP2 reporting checklists: tools to improve reporting of patient and public involvement in research. Res Involv Engagem. 2017;3(1):13.
18. Scholz B, Bevan A. Toward more mindful reporting of patient and public involvement in healthcare. Res Involv Engagem. 2021;7(1):61.
19. Boylan AM, Locock L, Thomson R, Staniszewska S. “About sixty per cent I want to do it”: Health researchers’ attitudes to, and experiences of, patient and public involvement (PPI)—A qualitative interview study. Health expectations : an international journal of public participation in health care and health policy. 2019;22(4):721-30.
20. Frank L, Forsythe L, Ellis L, Schrandt S, Sheridan S, Gerson J, et al. Conceptual and practical foundations of patient engagement in research at the patient-centered outcomes research institute. Quality of life research : an international journal of quality of life aspects of treatment, care and rehabilitation. 2015.
21. Boivin A, L'Espérance A, Gauvin F-P, Dumez V, Macaulay AC, Lehoux P, et al. Patient and public engagement in research and health system decision making: A systematic review of evaluation tools. Health expectations : an international journal of public participation in health care and health policy. 2018;21(6):1075-84.
22. Pollard K, Donskoy AL, Moule P, Donald C, Lima M, Rice C. Developing and evaluating guidelines for patient and public involvement (PPI) in research. Int J Health Care Qual Assur. 2015;28(2):141-55.
23. PCORI. Ways of Engaging- ENgagement ACtivity Tool (WE-ENACT) – Researcher 2.0 Item Pool2014 04.01.2016. Available from: http://www.pcori.org/sites/default/files/PCORI-WE-ENACT-Researcher-Survey-Item-Pool.pdf.
24. PCORI. Ways of Engaging- ENgagement ACtivity Tool (WE-ENACT) – Patients and Stakeholders 2.0 Item Pool2014 04.01.2016. Available from: http://www.pcori.org/sites/default/files/PCORI-WE-ENACT-Patient-Stakeholder-Survey-Item-Pool.pdf.
25. Stocks SJ, Giles SJ, Cheraghi-Sohi S, Campbell SM. Application of a tool for the evaluation of public and patient involvement in research. BMJ open. 2015;5(3):e006390.
26. Morrow E, Ross F, Grocott P, Bennett J. A model and measure for quality service user involvement in health research. Int J Consum Stud. 2010;34(5):532-9.
27. Abelson J, Li K, Wilson G, Shields K, Schneider C, Boesveld S. Supporting quality public and patient engagement in health system organizations: development and usability testing of the Public and Patient Engagement Evaluation Tool. Health Expect. 2016;19(4):817-27.
28. Hamilton CB, Hoens AM, McQuitty S, McKinnon AM, English K, Backman CL, et al. Development and pre-testing of the Patient Engagement In Research Scale (PEIRS) to assess the quality of engagement from a patient perspective. PloS one. 2018;13(11):e0206588.
29. Hamilton CB, Hoens AM, McKinnon AM, McQuitty S, English K, Hawke LD, et al. Shortening and validation of the Patient Engagement In Research Scale (PEIRS) for measuring meaningful patient and family caregiver engagement. Health expectations : an international journal of public participation in health care and health policy. 2021;24(3):863-79.
30. Abelson J, Tripp L, Kandasamy S, Burrows K, Team PIS. Supporting the evaluation of public and patient engagement in health system organizations: Results from an implementation research

For peer review only

study. Health expectations : an international journal of public participation in health care and health policy. 2019;22(5):1132-43.

31. Kearney A, Williamson P, Young B, Bagley H, Gamble C, Denegri S, et al. Priorities for methodological research on patient and public involvement in clinical trials: A modified Delphi process. Health expectations : an international journal of public participation in health care and health policy. 2017;20(6):1401-10.

32. Staniszewska S, Brett J, Mockford C, Barber R. The GRIPP checklist: strengthening the quality of patient and public involvement reporting in research. International journal of technology assessment in health care. 2011;27(4):391-9.

33. Staley K, Buckland SA, Hayes H, Tarpey M. 'The missing links': understanding how context and mechanism influence the impact of public involvement in research. Health expectations : an international journal of public participation in health care and health policy. 2014;17(6):755-64.

34. Domecq J, Prutsky G, Elraiyah T, Wang Z, Nabhan M, Shippee N, et al. Patient engagement in research: a systematic review. BMC health services research. 2014;14(1).

35. Snape D, Kirkham J, Preston J, Popay J, Britten N, Collins M, et al. Exploring areas of consensus and conflict around values underpinning public involvement in health and social care research: a modified Delphi study. BMJ open. 2014;4(1):e004217.

36. Boote J, Wong R, Booth A. 'Talking the talk or walking the walk?' A bibliometric review of the literature on public involvement in health research published between 1995 and 2009. Health expectations : an international journal of public participation in health care and health policy. 2015;18(1):44-57.

37. Biddle MSY, Gibson A, Evans D. Attitudes and approaches to patient and public involvement across Europe: A systematic review. Health Soc Care Community. 2021;29(1):18-27.

38. Schütt A, Müller-Fries E, Weschke S. Aktive Beteiligung von Patientinnen und Patienten in der Gesundheitsforschung–eine Heranführung für (klinisch) Forschende. Bonn/Berlin DLR Proj. 2023.

39. Schaefer I, Allweiss T, Dresen A, Amort FM, Wright MT, Krieger T. PartNet-Methodenpapier: Modell für Partizipative Gesundheitsforschung (PGF-Modell). 2022.

40. (BMBF) BfBuF. 2023.

41. Prinzipien für eine erfolgreiche Patientenpartizipation in der Krebsforschung. 2021.

42. Brütt AL, Borgmann S, Buchholz E, Burggraf L, Engler J, Fischer F, et al. DNVF Memorandum Partizipative Versorgungsforschung Teil 1. Gesundheitswesen. 2025.

43. Harrison JD, Auerbach AD, Anderson W, Fagan M, Carnie M, Hanson C, et al. Patient stakeholder engagement in research: a narrative review to describe foundational principles and best practice activities. Health Expectations. 2019;22(3):307-16.

44. Jackson T, Pinnock H, Liew SM, Horne E, Ehrlich E, Fulton O, et al. Patient and public involvement in research: from tokenistic box ticking to valued team members. BMC Med. 2020;18(1):79.

45. Levelink M, Voigt-Barbarowicz M, Brütt AL. Priorities of patients, caregivers and health-care professionals for health research - A systematic review. Health Expect. 2020;23(5):992-1006.

46. Bernges T, Iden L, Gielen R, Scholl M, Brütt AL. Forschen für uns! Welche Forschungsthemen interessieren von Depression Betroffene? Psychiatr Prax. 2018;45(7):383-6.

47. Brütt AL, Bernges T, Magaard J, Ex In Pilots, Sielaff G. Mitforschen, aber wie? Entwicklung und Evaluation eines Forschungstrainings fur Psychiatrieerfahrene. Psychiatr Prax. 2017;44(2):99-104.

48. Brütt AL, Meister R, Bernges T, Moritz S, Harter M, Kriston L, et al. Patient involvement in a systematic review: Development and pilot evaluation of a patient workshop. Z Evid Fortbild Qual Gesundhwes. 2017;127-128:56-61.

49. Levelink M, Eichstaedt HC, Meyer S, Brütt AL. Living with a left ventricular assist device: psychological burden and coping: protocol for a cross-sectional and longitudinal qualitative study. BMJ open. 2020;10(10):e037017.

50. Seeralan T, Härter M, Koschnitzke C, Scholl M, Kohlmann S, Lehmann M, et al. Patient involvement in developing a patient-targeted feedback intervention after depression screening in primary care within the randomized controlled trial GET.FEEDBACK.GP. Health expectations :

For peer review only

an international journal of public participation in health care and health policy. 2021;24 Suppl 1:95-112.
51. Ernstmann N, Heuser C, Pfaff H. Health services research facilities at German universities. Gesundheitswesen 2018;82(4):313-7.
52. Reynolds J, Ogden M, Beresford R. Conceptualising and constructing 'diversity' through experiences of public and patient involvement in health research. Res Involv Engagem. 2021;7(1):53.
53. Thompson J, Bissell P, Cooper C, Armitage CJ, Barber R. Credibility and the 'professionalized' lay expert: Reflections on the dilemmas and opportunities of public involvement in health research. Health. 2012;16(6):602-18.
54. Staley K, Elliott J, Stewart D, Wilson R. Who should I involve in my research and why? Patients, carers or the public? Res Involv Engagem. 2021;7(1):41.
55. Lander J, Langhof H, Dierks M-L. Involving patients and the public in medical and health care research studies: An exploratory survey on participant recruiting and representativeness from the perspective of study authors. PloS one. 2019;14(1):e0204187.
56. Mills T, Lawton R, Sheard L. Advancing complexity science in healthcare research: the logic of logic models. BMC medical research methodology. 2019;19:1-11.
57. Sammut R, Griscti O, Norman IJ. Strategies to improve response rates to web surveys: a literature review. International Journal of Nursing Studies. 2021;123:104058.
58. Harris PA, Taylor R, Thielke R, Payne J, Gonzalez N, Conde JG. Research electronic data capture (REDCap)-A metadata-driven methodology and workflow process for providing translational research informatics support. J Biomed Inform. 2009;42(2):377-81.
59. IBM SPSS Statistics for Windows. 29 ed. Armonk, NY: IBM Corp.; 2022.
60. Barbour RS. Doing focus groups. 2nd edition ed: SAGE; 2018.
61. Guest G, Namey E, McKenna K. How Many Focus Groups Are Enough? Building an Evidence Base for Nonprobability Sample Sizes. Field Methods. 2017;29(1):3-22.
62. Kuckartz U. Qualitative Inhaltsanalyse Methoden, Praxis, Computerunterstützung. 3., überarbeitete Auflage ed. Juventa V, editor: Beltz Juventa; 2016.
63. Software V. MAXQDA 2020. Berlin, Germany: VERBI Software; 2019.
64. Streiner DL. Health measurement scales a practical guide to their development and use. 5 ed ed. Norman GR, Cairney J, editors: Oxford University Press; 2015.
65. Mokkink LB, Terwee CB, Patrick DL, Alonso J, Stratford PW, Knol DL, et al. The COSMIN study reached international consensus on taxonomy, terminology, and definitions of measurement properties for health-related patient-reported outcomes. J Clin Epidemiol. 2010;63(7):737-45.
66. Wild D, Grove A, Martin M, Eremenco S, McElroy S, Verjee-Lorenz A, et al. Principles of Good Practice for the Translation and Cultural Adaptation Process for Patient-Reported Outcomes (PRO) Measures: report of the ISPOR Task Force for Translation and Cultural Adaptation. Value Health. 2005;8(2):94-104.
67. Miller K, Chepp V, Willson S, Padilla J-L. Cognitive interviewing methodology: John Wiley & Sons; 2014.
68. Polit DF, Beck CT, Owen SV. Is the CVI an acceptable indicator of content validity? Appraisal and recommendations. Research in Nursing & Health. 2007;30(4):459-67.
69. Terwee CB, Bot SD, de Boer MR, van der Windt DA, Knol DL, Dekker J, et al. Quality criteria were proposed for measurement properties of health status questionnaires. J Clin Epidemiol. 2007;60(1):34-42.
70. Frost MH, Reeve BB, Liepa AM, Stauffer JW, Hays RD, Group MFPROCM. What is sufficient evidence for the reliability and validity of patient-reported outcome measures? Value in Health. 2007;10:S94-S105.

For peer review only

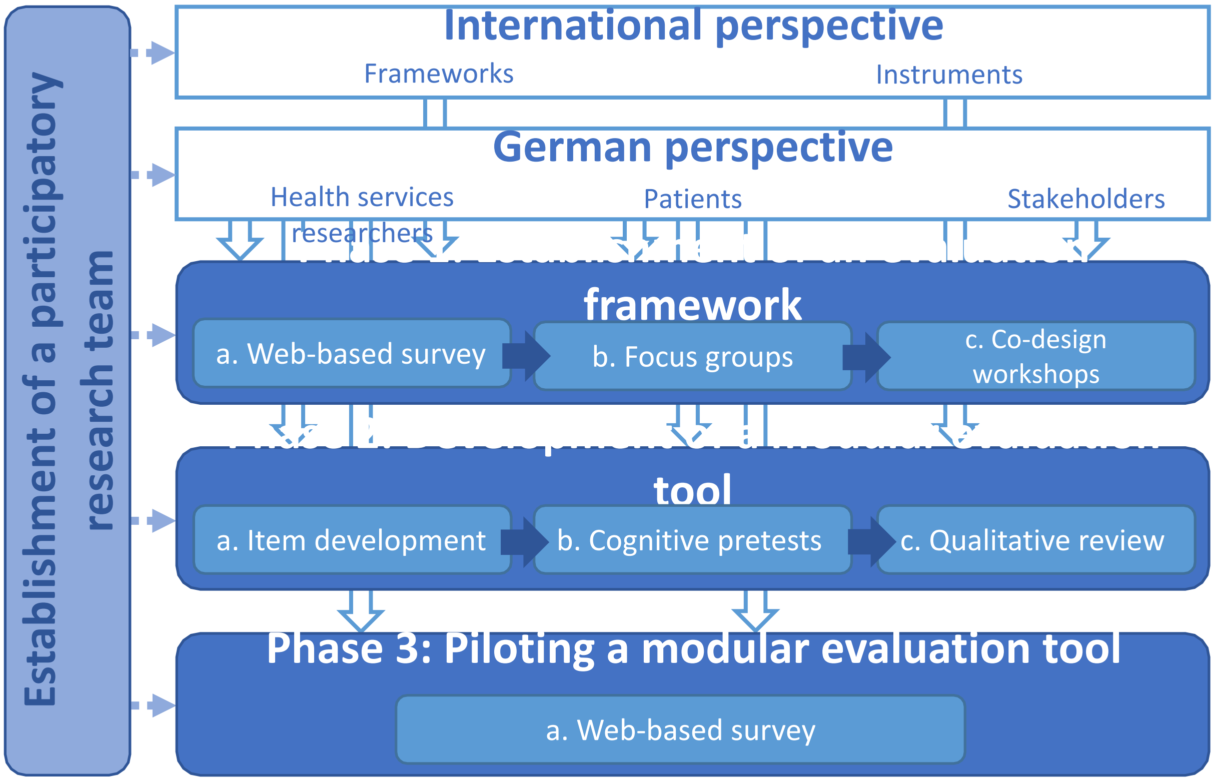

Figure 1. Overview of the study design.

BMJ Open

Development and Piloting of a Modular Evaluation Tool for Patient and Public Involvement in Health Services Research - Protocol of a Mixed-Methods Study

Journal:	*BMJ Open*
Manuscript ID	bmjopen-2025-110547.R1
Article Type:	Protocol
Date Submitted by the Author:	28-Oct-2025

Complete List of Authors:	Seeralan, Tharanya; University Medical Center Hamburg-Eppendorf, Department for Medical Psychology, Center for Psychosocial Medicine<br>Oster, Lena; University Medical Center Hamburg-Eppendorf, Department of Medical Psychology, Center for Psychosocial Medicine<br>Jones, Julia; University of Hertfordshire, Centre for Research in Public Health and Community Care, School of Health, Medicine and Life Sciences<br>Mathie, Elspeth ; University of Hertfordshire, Centre for Research in Public Health and Community Care, School of Health, Medicine and Life Sciences<br>Härter, Martin; University Medical Center Hamburg-Eppendorf, Department of Medical Psychology, Center for Psychosocial Medicine<br>Brütt, Anna Levke; University Medical Center Hamburg-Eppendorf, Department for Medical Psychology; Carl von Ossietzky University of Oldenburg School VI - Medicine and Health Sciences, Department of Health Services Research

**Primary Subject Heading**:	Health services research
Secondary Subject Heading:	Health services research
Keywords:	Patient Participation, Health Services, Community-Based Participatory Research, QUALITATIVE RESEARCH, Surveys and Questionnaires

SCHOLARONE™
Manuscripts

Development and Piloting of a Modular Evaluation Tool for Patient and Public Involvement in Health Services Research - Protocol of a Mixed-Methods Study

Tharanya Seeralan[1], Lena Oster[1], Julia Jones[2], Elspeth Mathie[2], Martin Härter[1], Anna Levke Brütt*[1, 3]

[1]Department of Medical Psychology, Center for Psychosocial Medicine, University Medical Center Hamburg-Eppendorf, Hamburg, Germany

[2] Centre for Research in Public Health and Community Care, School of Health, Medicine and Life Sciences, University of Hertfordshire, United Kingdom

[3] Department of Health Services Research, Junior Research Group for Rehabilitation Sciences, School of Medicine and Health Sciences, University of Oldenburg, Oldenburg, Germany

***Correspondence to:**

PD Dr. phil. Anna Levke Brütt, Dipl.-Psych., M. Sc.

Department of Medical Psychology

University Medical Center Hamburg-Eppendorf

Martinistraße 52, Building W26, 20246 Hamburg

Email address: a.bruett@uke.de

For peer review only

ABSTRACT

Background: There is limited evidence regarding the outcomes and impacts of Patient and public involvement (PPI) in research, mainly based on narrative studies. Existing frameworks for supporting and evaluating PPI often require adaptation to specific contexts, and comprehensive instruments are needed. From an international perspective, strengthening the scientific foundation that underpins PPI is crucial to generate stronger evidence to understand which approaches work best, in which contexts, and with what effects.

Objectives: To promote PPI implementation in German health research, this project aims to 1) establish an evaluation framework 2) develop a modular evaluation tool in form of a questionnaire, and 3) pilot and psychometrically validate the tool.

Methods and Analysis: A three-phase mixed methods approach will be employed, integrating qualitative and quantitative data. First, we will explore with researchers, research partners, and other stakeholders in health services research, what contributes to meaningful and successful PPI through a web-based survey and focus groups. Findings are discussed in a codesign workshop in which participants agree on an evaluation framework based on a LOGIC model. Second, items from international instruments that evaluate PPI are deductively assigned to the evaluation framework. Further items are developed based on the focus groups from phase 1. Cognitive pretests and qualitative review will be conducted with researchers and patients in order to refine the item pool and develop the evaluation tool. Third, the evaluation tool with modules for researchers and patients will be piloted in a web-based survey. Data analysis will include thematic analysis for qualitative data and descriptive and psychometric analyses for quantitative data. A participatory research team will provide ongoing support throughout all project phases.

Ethics and Dissemination: Ethical approval has been obtained from the Local Ethics Committee of the Center for Psychosocial Medicine, University Medical Center Hamburg-Eppendorf (LPEK-0889). The study will follow the principles of the Helsinki Declaration and good scientific practice. Results will be disseminated at national and international conferences, public symposiums, and in peer-reviewed journals, contributing to the internationally developing field of PPI in research and addressing relevant research gaps.

Keywords: Patient Participation, Community-Based Participatory Research, Health Services, QUALITATIVE RESEARCH, Surveys and Questionnaires

For peer review only

Strengths and limitations of this study

- This study employs a mixed methods approach, integrating qualitative (e.g., focus groups, cognitive pretests) and quantitative (e.g., surveys, psychometric validation) methods.
- It involves multiple stakeholders, including researchers, research partners, and patients, to ensure comprehensive insights and participatory development of the evaluation framework and modular tool.
- It addresses the lack of comprehensive frameworks for evaluating PPI in German health research, providing essential tools to support, report, and systematically assess the impacts of PPI.

For peer review only

BACKGROUND

Through patient and public involvement (PPI) in research, the knowledge, skills and expertise of those directly affected by health issues and/or research outcomes can be considered. Especially in the United Kingdom, the United States, and Canada, it is widely accepted that involving patients and the public in various roles[1] is possible at all stages of the research process.[2] They can prioritize research questions, assist during study implementation, review and interpret study results, and contribute to the dissemination of findings. [3] As a consequence, funders, policy-makers and research organizations increasingly expect patients to be involved, particularly in health services research.

There are specific national programs that support PPI by promoting and providing infrastructure. In the UK, support was initially provided by INVOLVE, a national advisory group funded by the UK government. INVOLVE has since been integrated into the National Institute for Health and Care Research (NIHR) and its functions are now part of broader NIHR initiatives. In the USA, the government-sponsored Patient-Centered Outcomes Research Institute (PCORI) strengthens patient and stakeholder relationships by building networks. Moreover, PCORI guides and supports PPI within the research it funds. In Canada, the Institutes for Health Research Strategy for Patient-Oriented Research (SPOR) acts as a funder and facilitator for PPI in research. Beneficial as well as challenging outcomes and impacts of PPI have been demonstrated. Systematic reviews[4] and umbrella reviews[5] suggest that researchers report a greater understanding and deeper insights into their research area as well as personal benefits such as motivation. Challenges include feeling uncomfortable sharing power over research and investing additional time in partnerships. [4 5] Patients, on the other hand, report benefits including increased knowledge and skills, a more positive attitude towards research, improved access to information, and personal benefits such as feeling empowered. However, patients also reported feelings of not being listened to or taken seriously by researchers, as well as feeling overwhelmed by tasks and responsibilities during the research process. [4 5] In terms of outcomes and impacts on the research process, PPI can lead to more patient-centered research questions. Moreover, the comprehensibility of patient information material, questionnaires and interview schedules may be improved. PPI also accounted for more appropriate recruitment strategies and enhanced implementation and dissemination.[6 7] Additionally, PPI may improve the health-related outcomes of the involved patients.[8] Challenges regarding the organization of research projects reflect the additional time required and the insufficient financing of PPI.[7 9] Most of the reported outcomes and impacts originate from narrative studies. As a consequence, the evidence-base in PPI concerning the outcomes, impact and good practice criteria is rather limited.[4 5 7 9 10]

There are several reasons why adequate evidence has not been generated thus far:

For peer review only

First, some researchers do not see a need to generate evidence on the outcomes and impacts of PPI, as they see it as a democratic right with intrinsic value.[10] The international debate on the need to measure the outcomes and impact of PPI is ongoing.[11] For example, Staley and Barron emphasize that PPI supports mutual learning between patients and researchers and does not require measuring its impact.[12] In contrast, Staniszewska points out that measuring outcomes and impacts strengthens the evidence base of PPI.[13] Boivin et al. further emphasized that meaningful involvement requires sound evaluation.[14] However, a compromise is that PPI evaluation should not be limited to measuring outcomes and impacts, but should also include the process and context.[10] Second, there is a heterogeneous terminology,[5] and a poor reporting of PPI,[7 8 15 16] limiting the detection of its outcomes and impacts. Recently, consensus-informed guidelines for reporting PPI in research (Guidance for Reporting Involvement of Patients and the Public; GRIPP2) have been published on the EQUATOR webpage (www.equator-network.org). The GRIPP2 includes minimum reporting requirements in a short form.[17] In addition to defining the aims related to PPI and the methods used, articles and reports should also include impacts and outcomes - positive as well as negative - along with their measurement. While challenges such as tokenistic PPI may still exist,[18] this improves the quality, transparency, and consistency of PPI reporting.[19] Third, there are numerous frameworks for supporting PPI in research.[11] A comprehensive review and synthesis of PPI frameworks identified five different types: power-focused, priority-setting, study-focused, report-focused and partnership-focused frameworks. It also revealed that most published frameworks are rarely used outside the groups that developed them. Therefore, the authors concluded that these frameworks are difficult to transfer to other contexts and require adaptation for specific settings.[11] Fourth, available tools for evaluating PPI do not take into account its complexity, as both the process and the context need to be described. Furthermore, outcomes and impacts on patients, researchers and the research itself need to be considered.[20]

There is an increasing number of tools available for evaluating PPI.[21] For example, the Public Involvement Impact Assessment Framework (PiiAF) aims to help researchers plan and assess the impacts of PPI. It compiled a set of questions on the values, approaches, focus, study design and impacts. PiiAF can assist in documenting the quality of PPI in research projects.[22] It can be tailored to specific projects, but there are no defined items. PCORI uses different templates to collect information about PPI from researchers[23] and patients[24]. Stocks et al.[25] developed an instrument to evaluate PPI from the patient's perspective focusing on the quality of involvement.[26] The instrument accounts for both personal (e.g., feeling valued, achieving goals, feeling empowered) as well as contextual factors (e.g., research participation, previous experience). Outcomes and impact on the research process are only implied.[26] The Public Patient Engagement Evaluation Tool [PPEET;[27]] evaluates its quality, including four principles: integrity of design and process, influence and impact, participatory culture,

For peer review only

collaboration and common purpose. Different versions for researchers and patients are available. Another Canadian instrument, the Patient Engagement In Research Scale (PEIRS), is one of the very few instruments that underwent psychometric evaluation.[28 29] The items reflect procedural requirements, convenience, contributions, team environment and interaction, support, feelings of being valued and benefits. While the PiiAF lacks standardization, the WE-ENACT tools do not account for the context. Similarly, the instrument by Stocks et al.[25] and the PPEET[27] fail to include information on how PPI is conducted. The PEIRS[28 29] does not encompass the perspective of researchers.

To date, none of these tools provides a comprehensive evaluation of PPI with regard to the context, process, outcomes or impacts from different perspectives. Moreover, their scientific rigor in the development and validation process requires improvement.[21] In summary, from an international perspective, there is a need to strengthen the science behind PPI to generate stronger evidence to understand which PPI approaches work best, in which contexts and with what effects.[16 21 30-35] In addition to these general methodological aspects, country-specific contexts must also be considered. The implementation of PPI is heterogeneous. For example, in Germany PPI has not yet been systematically implemented in health services research. Among the 683 studies (published up to 2009) included in the extensive review on PPI conducted by Boote et al.[36] only two originated from Germany. This has not changed over the past decade. In 2021, Biddle et al. concluded that the implementation of PPI is very heterogeneous and not yet firmly established or formalized in European health services research. They refer to a lack of infrastructure, guidance and support as potential barriers.[37]

Recently, German research funders have introduced PPI into health research as a requirement within project proposals, aiming to drive its practice. Meanwhile, some documents and principles have been published that provide orientation for participatory health research in Germany.[38-42] However, despite these important contributions, a comprehensive and systematic framework for evaluating PPI in health service research in Germany remains absent. The lack of an overarching evaluation concept may risk tokenistic approaches to PPI, as we have learned from international research.[19 43 44] Therefore, an evaluation framework for PPI is needed to adequately support, report and evaluate PPI[11] in health services research in Germany. Furthermore, while realizing PPI has been central in various of our previous studies,[45-50] we were also confronted with the lack of adequate instruments to evaluate the process, perceived impact, and potential harms of PPI. Thus far, there is a limited understanding of what constitutes meaningful and successful PPI from the perspectives of both researchers and patients in health services research in Germany.

OBJECTIVES

For peer review only

Therefore, the study objectives are (1) to establish a theoretical evaluation framework, (2) based on this, to develop a modular evaluation tool as a practical questionnaire for various stakeholders and (3) to pilot the modular evaluation tool including psychometric testing.

We would like to prepare an evaluation framework for PPI in health services research in Germany, considering the context, process, outcomes and impacts. The objective of establishing an evaluation framework is related to the following research questions:

1. *What does meaningful and successful involvement in health services research look like for researchers and patients in Germany?*
2. *How should the evaluation of PPI be approached in health services research in Germany?*

We will acknowledge the international literature and will explore the specifics of PPI in health services research in Germany. Items will be devised acknowledging existing instruments as well as qualitative survey and interview data. Related research questions are:

3. *Do the devised items address the consented evaluation framework?*
4. *Are the devised items of the modular evaluation tool comprehensive and relevant?*

The developed modular evaluation tool will undergo validity and reliability testing.

5. *Does the modular evaluation tool produce stable and consistent results?*

We will place further emphasis on PPI in the evaluation design and reporting by involving a participatory research team in all phases of the research process.

METHODS AND ANALYSIS

Study design

The proposed 3-year mixed-methods project comprises three phases. Each phase examines one of the objectives described above (figure 1).

– please insert "Figure 1. Overview of the study design" about here –

Study population

The study will focus on the perspectives of researchers, patients, and stakeholders with PPI experience within the context of German health services research. Adult patients (≥ 18 years) with lived experience in treatment-relevant health conditions and experience with the healthcare system will be included. Patients with limited proficiency in the German language, as well as those with severe impairments (e.g., cognitive impairment or lower quality of life due to illness), will be excluded. Researchers and patients will be recruited through databases on publicly funded health services research and via recently established PPI networks (e.g., DNVF patient advisory board, personal

For peer review only

contacts). Detailed information regarding specific study population and recruitment strategy will be provided aligning with the description of the three phases and work packages presented below.

Relevance of sex, gender and/or diversity

Special attention will be given to considering the diverse perspectives of researchers and patients. In web-based surveys, researchers and patients with relevant experiential knowledge about patient involvement can contribute. The community of health services researchers in Germany is heterogeneous,[51] but diversity has not been investigated thus far. Patients involved in this research are often white, female and retired.[52] It is therefore likely that, in aiming to engage individuals and community groups already experienced in participatory research, participating patients may have a narrow set of sociodemographic characteristics, and the views of a more diverse population may be underrepresented. We will include diversity aspects in questions regarding sociodemographic characteristics and will therefore be able to at least describe them in the survey sample. Setting up a web-based survey will not produce selection bias among researchers. However, patients who have previously been involved in research will presumably be familiar with the technique.

For the qualitative study parts, we will stratify our sampling strategies based on relevant sociodemographic aspects to reflect diversity. We will sample researchers with regard to gender, age/research stage, and the field of expertise/research methods. With regard to patients, we consider gender, age and education to be relevant. In addition, we will include patients with different health conditions and associated networks (individual patient vs. member/representative in a patient organization). By asking for sociodemographic characteristics, we will be able to report on diversity in the qualitative study parts.

Moreover, we aim to identify a diverse group of people, by gender, age and educational background, to join the PPI group that we are calling the participatory research team. This team should also include experience with different kinds of diseases and networks as well as experience with being a patient partner. On the one hand, we assume that patient partners acquire knowledge that is useful for future participatory research teams.[53 54] On the other hand, we will recruit additional persons without prior experience in PPI. We assume that skills such as confidence in articulating oneself in a formal meeting would support an interest in joining a participatory research team. As suggested by Thompson et al.[53] and Lander et al.[55], we will reflect on which input will be most useful, provide reasons for why certain participants were selected and discuss how potential recruiting and representativeness limitations impact the process and results.

Phase 1: Establishing an evaluation framework

Aim and methods

For peer review only

We aim to contribute to a scientifically sound, comprehensive evaluation framework for PPI in health services research in Germany based on a logic model.[56] It will be used to describe the causal processes through which PPI produces outcomes and impacts. Information on meaningful and successful PPI in health services research from the perspective of researchers and patients will be gathered by conducting a web-based survey and focus group interviews. Results will be presented in a co-design workshop to establish an evaluation framework (see figure 1).

a. Web-based survey

The web-based survey consists of different modules for researchers and patients. They will be recruited through several time-shifted strategies: a) identification and invitation of principal investigators of publicly funded health services research, b) identification and invitation of researchers and patients (or their representatives) involved in publicly funded health services research that implements PPI, and c) through personal knowledge (e.g., Deutsches Netzwerk Versorgungsforschung (DNVF), including its patient advisory board).

Recruitment and procedures

Principal investigators of publicly funded projects in the field of health services research (German Research Foundation (Deutsche Forschungsgemeinschaft, DFG), Federal Ministry of Research, Technology and Space (Bundesministerium für Forschung, Technologie und Raumfahrt, BMFTR), Federal Ministry of Health (Bundesministerium für Gesundheit, BMG), innovation fund of The Federal Joint Committee (Gemeinsamer Bundesausschuss, G-BA) can be identified using databases (e.g., funded projects information system (GEPRIS), funding catalogue of the BMFTR). The inclusion criteria specify that the project must be conducted in Germany, initiated in 2010 or later, and aimed to gain knowledge about healthcare. In the GEPRIS Database currently lists 78 researchers, while the BMBF funding catalogue contains 571 projects. Researchers will be contacted via email and invited to participate in a linked web-based survey. With regard to sampling strategy 2, these principal investigators can name further researchers and patients for participation in the survey. Four weeks after the second reminder email has been sent to the principal investigators, sampling strategies b) and c) will be applied. Links to the web-based survey will be sent via email. Two reminders will be sent.[57] The web application Research electronic data capture (REDCap) will be used to implement the web-based survey.[58] Informed consent will be obtained online.

In module B, all participating principal investigators and researchers and patients will provide information on individual characteristics. Researchers will answer questions on age, gender, seniority[19] and patients will be asked questions with regard to age, gender, health condition with lived experience and membership of self-help organization. In module C, the participants are presented with information on PPI and will be asked to rate different elements of the frameworks for supporting PPI in research according to their relevance.[11 38-41] For both aspects, a nine-point Likert scale (1=not

For peer review only - http://bmjopen.bmj.com/site/about/guidelines.xhtml

relevant; 9=very relevant) will be used. In addition, open-ended questions will be asked about whether any relevant aspects are missing. At the end of the web-based survey, participants can consent to be contacted for further interviews or surveys in the project, which requires storing their email address and further details (such as project and investigator characteristics) in a project database and/or for receiving information about the project results.

Data analysis

To analyse the project and participant characteristics, we will calculate the median, mean and standard deviation (SD) for the interval data and frequencies for the nominal items. In the analysis of the ratings (module C), the median, mean and SD will be calculated. All data will be analysed using IBM SPSS Statistics 29.[59] The descriptive analysis of the web survey is intended to provide an overview of the relevant domains of internationally developed PPI frameworks from the perspective of health services researchers in Germany.

b. Focus groups

Focus groups[60] will be conducted to validate and expand the findings of the web-based survey. Researchers as well as patients experienced in PPI will be recruited. An interview guide will be developed based on the different elements of the logic framework and will be adapted for researchers and patients. The conduction of focus groups with patients will be discussed and planned together with the participatory research team.

Recruitment and procedures

Six focus groups will take place, separately with researchers and patients, with 5 to 7 participants moderated by two members of the project team. Empirical studies have shown that 3 to 6 focus groups will be enough to discover 90% of the themes.[61] We will recruit participants as outlined. We aim to include a broad range of researchers and patients experienced with PPI in health services research. We will aim for maximum variation in the researcher sample with regard to early-stage and advanced researchers, different fields of expertise (e.g., mental health services research) and diversity with regard to individual participants (e.g., gender). The patient sample is planned to include diversity with regard to health conditions and associated networks (e.g., individual patients vs. member/representative in/of patient organization) as well as sociodemographic factors (e.g., gender, age, educational background). The interview guide will be tailored to the participant groups. The core questions concern the context, process, outcomes and impact of PPI. A detailed interview guide will be developed involving the participatory research team.

Data analysis

The focus group interviews will be audio-recorded and transcribed verbatim by a professional transcription company. Afterwards, qualitative thematic analysis[62] will be applied. Relevant passages

For peer review only

will be coded using a deductive category system derived from the interview guide. Furthermore, inductive coding to refine the main categories will be applied. There will be two coders, and intercoder reliability will be assessed before the consensus discussions take place within the project team. For the documentation and analysis of the data, the software MAXQDA 2020[63] will be used.

c. Codesign workshop

The codesign workshop is planned according to the suggestions of Greenhalgh and colleagues.[11] It will be carried out with approximately 16 researchers, patients and further relevant stakeholders (e.g., representatives of the funding bodies or health insurances) in the field of health services research in Germany.

Recruitment, procedures and data analysis

Researchers and patients who consented to participate in the previous study parts will be considered; written informed consent will be obtained during each focus group and codesign workshop via paper-pencil. At least four persons from each group should participate in the codesign workshop. Criteria with regard to diversity, only gender and age, will be applied due to the low number of participants in the codesign workshop. Stakeholders should reflect the field of health services research, e.g., representatives of the funding bodies or health insurance. The codesign workshop will be a whole day workshop (6 hours). It will start in plenary to present the condensed results of the web-based survey and the themes identified in the focus groups. Participants will have the opportunity to ask questions and to discuss the themes. Following the suggestions,[11] each participant will be asked to choose up to 10 themes that they think are the most important as indicated by multichoice voting. Participants can indicate their preferences by marking points at the respective themes, which are displayed on flip charts. Accordingly, votes are collected from all participants and will be available in real-time. This information will be available for the following group work aiming at relating the identified themes to the logic model. Participants will work in four groups of approximately four people. Each group should include a mix of researchers, patients and further stakeholders. Each group will be assigned to one of the domains of the logic model (context, process, outcome and impact). Participants can use the presented themes and choose those that reflect the respective domain of the logic model. Afterwards, the preliminary version of the evaluation framework will be presented and discussed in plenary. In particular, themes that have been chosen by more than one group will be discussed and specified. Then, participants will return to the groups to refine their domain based on the discussions. Again, the group will present their domain. The process is repeated if participants bring up further discussion points. It is possible that the evaluation framework will include different domains for researchers and patients. Finally, the framework as a whole will be discussed in plenary and accepted in consensus.

Phase 2: Development of a modular evaluation tool

For peer review only - http://bmjopen.bmj.com/site/about/guidelines.xhtml

Aim and methods

The development of the modular evaluation tool in the form of a questionnaire is based on Streiner et al.'s[64] recommendations, including devising and reviewing items. Aspects of validity will also be considered.[65]

a. Item development

In the first step, we generate an item pool based on the existing instruments. In the second step, we will use the themes developed within the analysis of the free-text answers in the web-based survey and focus groups to develop additional items. As numerous instruments have already been developed internationally, as displayed in the review by Boivin et al.[21], we will acknowledge these by linking their items to the developed framework.

Procedures and data analysis

Since PPI is a dynamic field and several instruments have not been included[28 29] in a previous systematic review,[21] we will first update the review alongside the data collection during the first phase of the project (see figure 1). Afterwards, the items of all identified instruments will be systematically collected. Then, qualitative thematic analysis[62] will be applied using a deductive category system derived from the components of the consented evaluation framework (see phase 1). Each item will be linked to the content areas. The results will be displayed in a matrix.[64] There will be two coders, and intercoder reliability will be assessed before consensus discussions will follow. For the documentation and analysis of the data, the computer program MAXQDA 2020[63] will be used. Second, items from existing questionnaires for PPI evaluation that match the evaluation framework will undergo a standardized translation procedure, including forwards and backwards translations[66]. Additionally, the research team will formulate additional items based on the themes derived from the free-text answers of the web-based survey and the focus groups. Ideally, the phrasing of the items is close to the original the statements of the participants. This process will result in a comprehensive item pool and the next step includes eliminating redundant or poorly worded items. An iterative process including the participatory research team will be used to refine the items. Afterwards, scaling will be specified. Finally, a preliminary version that undergoes cognitive pretesting will be defined. With regard to the proposed modular character of the evaluation tool, separate modules can be developed for researchers and patients.

b. Cognitive pretests

Procedures and data analysis

Cognitive pretests will use the 'Think Aloud' technique.[67] We will invite 5 researchers and 5 patients who have already participated in the previous study phases to the first round; consent will be obtained on-site using paper-pencil forms. The researchers and patients will be asked to verbalize their thoughts

For peer review only

that lead them to the answer while filling out their questionnaires. We plan to conduct cognitive pretests in person. Therefore, we will primarily recruit patients locally. The 'Think Aloud' will be recorded in writing and evaluated. Using the data from the cognitive pretests, the researchers and the participatory research team will discuss and decide upon revisions. The decisions will be reached by consensus. The revised items will be presented in a second round of cognitive pretests involving another 5 researchers and 5 patients. After a further revision, the pretested and revised modular evaluation tool will undergo qualitative review.

c. Qualitative review

Procedures and data analysis

An online qualitative review will be conducted to investigate the face validity of the developed items. Researchers, patients and stakeholders who participated in the codesign workshop will be invited for the qualitative review. A web-based survey including all items will be administered using REDCap; informed consent will be obtained online.[58] Participants will judge each item with regard to its relevance on a 4-point scale (1: not relevant, 2: somewhat relevant, 3: quite relevant, 4: highly relevant). They will also be asked whether any important questions are missing. To ensure that all items are relevant and reflect the evaluation framework, the Content Validity Index (CVI) will be calculated.[68] The CVI is computed as the number of experts giving a rating of "very relevant" for each item divided by the total number of experts. Values range from 0 to 1 where CVI > 0.79, the item is relevant; between 0.70 and 0.79, the item needs revisions; and if the value is below 0.70, the item is eliminated. Nevertheless, there should be at least three items per domain. Based on the scores, a revision will be made.

Phase 3: Piloting a modular evaluation tool

Aim and methods

The pilot study is intended to provide information on the reliability and validity of the developed modular evaluation tool. We will consider the relevant literature on the quality criteria for health outcome measurements.[65 69] With regard to reliability, we will focus on internal consistency and retest reliability, as there are no measurement standards for PPI evaluation tools. Additionally, we will account for the additional aspects of construct validity: factorial validity and convergent validity.

a. Web-based pilot study

Recruitment and procedures

Researchers and patients will be recruited by updating identifying the principal investigators of ongoing publicly funded health services research. Additionally, snowball sampling and sampling through personal knowledge will be applied. Researchers and patients will be invited to take part in

web-based pilot testing of the modular evaluation tool. They will receive emails and individualised links. Reminders will be sent to improve the response rates.[57] Participants are eligible when they are involved in a currently ongoing health research project that implements PPI. At the end of each survey, participants can also generate a code for specific ongoing projects and send links to the web-based pilot study to identify other project members. The code can be used to merge evaluations on specific projects. Moreover, participants can choose whether they would like to receive a score sheet after closure of the pilot study.

Overall, the pilot study is intended to collect a total of at least 200 responses. Modules that can be answered by researchers or patients only should be answered by at least 100 participants. This will constitute an appropriate sample size providing insights into psychometric testing.[70] A randomly chosen subsample of n=50 will be invited to fill in the core modules a second time after approximately 1 week. REDCap[58] will be used to implement the web-based survey. Informed consent will be obtained online.

The survey will include the items from the developed modular evaluation tool items as well as the PPEET[27] to establish convergent validity. The PPEET has previously been used for validation studies,[29] and an authorized German version of the PPEET exists. Participating researchers and patients will also be asked to provide information on individual characteristics such as age and gender, as well as seniority for researchers and patients' health condition with lived experience for patients, respectively.

Data analysis

For sample characteristics, we will consider descriptive data. For item analysis, we will use descriptive data (N, mean, standard deviation, range, skewness, kurtosis and item difficulty). Internal consistency will be evaluated by Cronbach's α or the Kuder Richardson coefficient for dichotomous items. To assess test-retest reliability and construct validity, we will calculate correlations. We assume moderate correlations of the modules with related items of the PPEET. With regard to factorial validity, we will perform exploratory factor analysis. Further relevant aspects for reliability and validity testing, e.g., additional scales to be used and strengths of correlation, will be determined when the evaluation framework is agreed upon. All data will be analysed using SPSS[59]. The aim is to obtain a modular tool with internally consistent scales (Cronbach's $\alpha > 0.70$) that captures a broad spectrum of factors relevant for PPI as identified in the evaluation framework.

Patient and public involvement

This researcher-initiated study was discussed with participatory research teams and patient advisory boards of previous and ongoing studies. For the proposed three-year project, a participatory research team comprising eight patient partners will be set up. The first meetings will be used to offer a research

For peer review only

training to all patient partners. During the different phases of the project, they will for example, give feedback on study information and consent forms for the web-based survey, the patient focus groups and the codesign workshop. They will assist in recruiting strategies, discussing qualitative analysis, evaluating cognitive pretests and discussing the proposed findings of this study's dissemination of project progress and results. Further involvement can be agreed on during the research process. They will assist in writing papers by reviewing early drafts and providing feedback. We plan to involve the participatory research team in all phases of the project through in-person and virtual meetings. Additional feedback from the participatory research team can be obtained via email between the meetings for specific aspects. Members of the participatory research team will be offered an expense allowance to acknowledge their contributions. The participatory process will be continuously evaluated through short surveys after each meeting and systematic reflections on process aspects e.g., team interactions, changes and impacts.[26 27]

ETHICS AND DISSEMINATION

Ethical considerations

Researchers and patients will participate in the study. This study does not involve any medical or psychological intervention that poses risk or harm to participants. The study will be conducted in accordance with the principles of the Helsinki Declaration and the standards of good scientific practice. All participants will be informed about the meaning, purpose and procedure of the study as well as the handling of the collected data. Informed consent will be obtained from all participants prior to taking part in the study. Approval from the Local Ethics Committee of the Center for Psychosocial Medicine, University Medical Center Hamburg-Eppendorf (LPEK-0889) and data security officer has been be obtained (LPEK-0889).

Data handling

New data will be generated in the empirical parts of the proposed project (survey, interviews). Researchers and patients will provide quantitative (e.g., ratings) as well as qualitative data (e.g., text, speech) in the web-based surveys, focus groups and co-design workshops. The web-based surveys conducted in phase 1 and phase 3, along with the qualitative review, will use the REDCap. REDCap supports data collection in accordance with the law (i.e., DGSVO), protecting the privacy of respondents (e.g., secure infrastructure, no cookies, no IP addresses in log files). For transcription of the focus groups, audio recordings will be uploaded to a secure and encrypted network and handled, ensuring data protection regulations. Quality checks of the transcripts will be performed by the interviewers and, if agreed on, by the interviewees. Audio recordings will be deleted after

For peer review only

transcription, and the transcripts will be anonymised. Protocols will be made available for those who took park in the workshops or cognitive debriefings. This allows a quality check.

Informed consent for participation in the online surveys, focus groups, co-design workshops, and cognitive debriefing, and qualitative review will be obtained. The project team will store all data on a secure and encrypted network drive, with access to project team members. Declarations of consent and transcripts or protocols will be archived separately for 10 years before deletion. When compiling the results, no information will be published that could allow conclusions to be drawn about individual persons.

Dissemination plan

Attention will be given to effectively disseminating project results to researchers and patients. In accordance with open science practices, study deliverables, especially the evaluation framework and the evaluation tool, will be licensed under a creative commons license and made available on the project website. For both deliverables, patient-friendly accompanying material developed with the participatory research team will be available. After piloting of the modular evaluation tool, it is aimed for providing an online tool that allows researchers and patients to evaluate PPI in specific projects. This holds the opportunity to monitor of PPI in health services research in Germany, but also to enable researchers and patients to receive individual feedback. The implementation of the online evaluation tool will be envisaged for a second funding period.

The study results will be presented to the participating researchers and patients in a public symposium. disseminated at national and international conferences and published in peer-reviewed journals. Moreover, there will be open access to the scientific results (e.g., open access articles), and we will provide lay-friendly summaries of published scientific articles on the project website. As is customary, the results will be reported back to the funder upon project completion.

Status of the study

The study started on January 1, 2025, data collection is planned to start on September 22, 2025 and the project is scheduled for completion on December 31, 2027 according to the current timeline. To date, approval from the Local Ethics Committee of the Center for Psychosocial Medicine, University Medical Center Hamburg-Eppendorf (LPEK-0889) has been obtained, we have started contacting interested patient partners for the participatory research team and initiated collaborations with international colleagues and cooperation partners. No substantive work within the different study phases has begun prior to the publication of this study protocol.

For peer review only

Statements

Declaration of Interest

The authors declare that they have no conflict of interest.

Tharanya Seeralan ORCID: 0000-0003-4431-0310
Lena Oster ORCID: 0009-0006-4744-2393
Elspeth Mathie ORCID: 0000-0002-5871-436X
Julia Jones ORCID: 0000-0003-3221-7362
Martin Härter ORCID: 0000-0001-74443-9890
Anna Levke Brütt ORCID: 0000-0002-4197-6048

Funding

This study was funded by The German Research Foundation (in German: Deutsche Forschungsgemeinschaft, DFG) under grant number 511611780. The sponsor had no influence on study design, data collection, management and analysis as well as interpretation of data, writing or publication process.

Author Contributions

ALB is the principal investigator who designed the study, wrote the grant proposal, and obtained the funding. ALB, TS, LO, JJ, EM and MH further refined the study by conceptualizing the research design, working out study processes, and coordinating the engagement of interested patient partners and associations. ALB and TS created the first draft of the manuscript. All authors contributed to critical revision of the manuscript for important intellectual content and approved the final version of the manuscript for publication.

Transparency Declaration

The corresponding author and manuscript guarantor [ALB] affirms that the manuscript is an honest, accurate, and transparent account of the study being reported, that no important aspects of the study have been omitted and that any discrepancies from the study registration will be reported.

Data Availability

The data that support the findings of this study will be available on reasonable request from the corresponding author [ALB].

For peer review only

Analytic Code Availability

The analytic code associated with this manuscript will be available on reasonable request from the corresponding author [ALB], only for purposes of reproducing results or replicating procedures.

Research Material Availability

The research materials associated with this manuscript will be available on reasonable request from the corresponding author [ALB], only for purposes of reproducing results or replicating procedures.

Figure Legends

Figure 1. Overview of the study design”

REFERENCES

1. Bird M, Ouellette C, Whitmore C, et al. Preparing for patient partnership: A scoping review of patient partner engagement and evaluation in research. *Health Expect* 2020;23(3):523-39. doi: 10.1111/hex.13040
2. Forsythe LP, Ellis LE, Edmundson L, et al. Patient and stakeholder engagement in the PCORI pilot projects: description and lessons learned. *Journal of general internal medicine* 2016;31:13-21.
3. Forsythe L, Heckert A, Margolis MK, et al. Methods and impact of engagement in research, from theory to practice and back again: early findings from the Patient-Centered Outcomes Research Institute. *Quality of Life Research* 2018;27(1):17-31. doi: 10.1007/s11136-017-1581-x
4. Brett J, Staniszewska S, Mockford C, et al. A systematic review of the impact of patient and public involvement on service users, researchers and communities. *Patient* 2014;7(4):387-95. doi: http://dx.doi.org/10.1007/s40271-014-0065-0
5. Hoekstra F, Mrklas KJ, Khan M, et al. A review of reviews on principles, strategies, outcomes and impacts of research partnerships approaches: a first step in synthesising the research partnership literature. *Health Res Policy Syst* 2020;18(1):51. doi: 10.1186/s12961-020-0544-9
6. McCarron TL, Clement F, Rasiah J, et al. Patients as partners in health research: A scoping review. *Health Expect* 2021;24(4):1378-90. doi: 10.1111/hex.13272
7. Brett J, Staniszewska S, Mockford C, et al. Mapping the impact of patient and public involvement on health and social care research: a systematic review. *Health Expect* 2014;17(5):637-50. doi: 10.1111/j.1369-7625.2012.00795.x
8. Halvorsrud K, Kucharska J, Adlington K, et al. Identifying evidence of effectiveness in the co-creation of research: a systematic review and meta-analysis of the international healthcare literature. *J Public Health (Oxf)* 2021;43(1):197-208. doi: 10.1093/pubmed/fdz126

For peer review only

9. Snape D, Kirkham J, Britten N, et al. Exploring perceived barriers, drivers, impacts and the need for evaluation of public involvement in health and social care research: a modified Delphi study. *BMJ Open* 2014;4(6):e004943. doi: 10.1136/bmjopen-2014-004943

10. Russell J, Fudge N, Greenhalgh T. The impact of public involvement in health research: what are we measuring? Why are we measuring it? Should we stop measuring it? *Res Involv Engagem* 2020;6:63. doi: 10.1186/s40900-020-00239-w

11. Greenhalgh T, Hinton L, Finlay T, et al. Frameworks for supporting patient and public involvement in research: Systematic review and co-design pilot. *Health Expect* 2019;22(4):785-801. doi: 10.1111/hex.12888

12. Staley K, Barron D. Learning as an outcome of involvement in research: what are the implications for practice, reporting and evaluation? *Research Involvement and Engagement* 2019;5(1):14. doi: 10.1186/s40900-019-0147-1

13. Staniszewska S, Herron-Marx S, Mockford C. Measuring the impact of patient and public involvement: the need for an evidence base. *Int J Qual Health Care* 2008;20(6):373-4. doi: 10.1093/intqhc/mzn044

14. Boivin A, Richards T, Forsythe L, et al. Evaluating patient and public involvement in research. *Bmj* 2018;363:k5147. doi: 10.1136/bmj.k5147

15. Mathie E, Wilson P, Poland F, et al. Consumer involvement in health research: A UK scoping and survey. *Int J Consum Stud* 2014;38(1):35-44. doi: 10.1111/ijcs.12072

16. Shippee ND, Domecq Garces JP, Prutsky Lopez GJ, et al. Patient and service user engagement in research: a systematic review and synthesized framework. *Health Expect* 2015;18(5):1151-66. doi: 10.1111/hex.12090

17. Staniszewska S, Brett J, Simera I, et al. GRIPP2 reporting checklists: tools to improve reporting of patient and public involvement in research. *Res Involv Engagem* 2017;3(1):13. doi: 10.1186/s40900-017-0062-2

18. Scholz B, Bevan A. Toward more mindful reporting of patient and public involvement in healthcare. *Res Involv Engagem* 2021;7(1):61. doi: 10.1186/s40900-021-00308-8

19. Boylan AM, Locock L, Thomson R, Staniszewska S. “About sixty per cent I want to do it”: Health researchers’ attitudes to, and experiences of, patient and public involvement (PPI)—A qualitative interview study. *Health Expect* 2019;22(4):721-30.

20. Frank L, Forsythe L, Ellis L, et al. Conceptual and practical foundations of patient engagement in research at the patient-centered outcomes research institute. *Quality of life research : an international journal of quality of life aspects of treatment, care and rehabilitation* 2015 doi: 10.1007/s11136-014-0893-3

21. Boivin A, L'Espérance A, Gauvin F-P, et al. Patient and public engagement in research and health system decision making: A systematic review of evaluation tools. *Health Expect* 2018;21(6):1075-84. doi: 10.1111/hex.12804

22. Pollard K, Donskoy AL, Moule P, et al. Developing and evaluating guidelines for patient and public involvement (PPI) in research. *Int J Health Care Qual Assur* 2015;28(2):141-55. doi: http://dx.doi.org/10.1108/IJHCQA-01-2014-0001

For peer review only

23. PCORI. Updated Engagement Plan Template. 2025. https://www.pcori.org/sites/default/files/PCORI-Updated-Engagement-Plan-Template.pdf (accessed 04.10.2025).
24. PCORI. Ways of Engaging- ENgagement ACtivity Tool (WE-ENACT) – Patients and Stakeholders 3.0 Item Pool. 2016. http://www.pcori.org/sites/default/files/PCORI-WE-ENACT-Patient-Stakeholder-Survey-Item-Pool.pdf (accessed 04.10.2025).
25. Stocks SJ, Giles SJ, Cheraghi-Sohi S, Campbell SM. Application of a tool for the evaluation of public and patient involvement in research. *BMJ Open* 2015;5(3):e006390. doi: 10.1136/bmjopen-2014-006390
26. Morrow E, Ross F, Grocott P, Bennett J. A model and measure for quality service user involvement in health research. *Int J Consum Stud* 2010;34(5):532-39. doi: 10.1111/j.1470-6431.2010.00901.x
27. Abelson J, Li K, Wilson G, et al. Supporting quality public and patient engagement in health system organizations: development and usability testing of the Public and Patient Engagement Evaluation Tool. *Health Expect* 2016;19(4):817-27. doi: 10.1111/hex.12378
28. Hamilton CB, Hoens AM, McQuitty S, et al. Development and pre-testing of the Patient Engagement In Research Scale (PEIRS) to assess the quality of engagement from a patient perspective. *PloS one* 2018;13(11):e0206588.
29. Hamilton CB, Hoens AM, McKinnon AM, et al. Shortening and validation of the Patient Engagement In Research Scale (PEIRS) for measuring meaningful patient and family caregiver engagement. *Health Expect* 2021;24(3):863-79.
30. Abelson J, Tripp L, Kandasamy S, et al. Supporting the evaluation of public and patient engagement in health system organizations: Results from an implementation research study. *Health Expect* 2019;22(5):1132-43. doi: 10.1111/hex.12949
31. Kearney A, Williamson P, Young B, et al. Priorities for methodological research on patient and public involvement in clinical trials: A modified Delphi process. *Health Expect* 2017;20(6):1401-10. doi: 10.1111/hex.12583
32. Staniszewska S, Brett J, Mockford C, Barber R. The GRIPP checklist: strengthening the quality of patient and public involvement reporting in research. *International journal of technology assessment in health care* 2011;27(4):391-9. doi: 10.1017/S0266462311000481
33. Staley K, Buckland SA, Hayes H, Tarpey M. 'The missing links': understanding how context and mechanism influence the impact of public involvement in research. *Health Expect* 2014;17(6):755-64. doi: 10.1111/hex.12017
34. Domecq J, Prutsky G, Elraiyah T, et al. Patient engagement in research: a systematic review. *BMC health services research* 2014;14(1) doi: 10.1186/1472-6963-14-89
35. Snape D, Kirkham J, Preston J, et al. Exploring areas of consensus and conflict around values underpinning public involvement in health and social care research: a modified Delphi study. *BMJ Open* 2014;4(1):e004217. doi: 10.1136/bmjopen-2013-004217
36. Boote J, Wong R, Booth A. 'Talking the talk or walking the walk?' A bibliometric review of the literature on public involvement in health research published between 1995 and 2009. *Health Expect* 2015;18(1):44-57. doi: 10.1111/hex.12007

For peer review only

37. Biddle MSY, Gibson A, Evans D. Attitudes and approaches to patient and public involvement across Europe: A systematic review. *Health Soc Care Community* 2021;29(1):18-27. doi: 10.1111/hsc.13111

38. Schütt A, Müller-Fries E, Weschke S. Aktive Beteiligung von Patientinnen und Patienten in der Gesundheitsforschung–eine Heranführung für (klinisch) Forschende. *Bonn/Berlin DLR Proj* 2023

39. Schaefer I, Allweiss T, Dresen A, et al. PartNet-Methodenpapier: Modell für Partizipative Gesundheitsforschung (PGF-Modell). 2022

40. Bundesministerium für Bildung und Forschung. Partizipationsstrategie Forschung. 2023. https://www.bmftr.bund.de/SharedDocs/Downloads/DE/2023/partizipationsstrategie.pdf?__blob=publicationFile&v=4 (accessed 04.10.2025)

41. Nationale Dekade gegen Krebs. 2021. Prinzipien für eine erfolgreiche Patientenpartizipation in der Krebsforschung. 2021. https://www.dekade-gegen-krebs.de/SharedDocs/Downloads/de/files/prinzipien-fuer-eine-erfolgrei-n-in-der-krebsforschung_web_bf.pdf?__blob=publicationFile&v=5. (accessed 04.10.2025)

42. Brütt AL, Borgmann S, Buchholz E, et al. DNVF Memorandum Partizipative Versorgungsforschung (Teil 1). *Gesundheitswesen* 2025 doi: 10.1055/a-2665-0028 [published Online First: 2025/10/27]

43. Harrison JD, Auerbach AD, Anderson W, et al. Patient stakeholder engagement in research: a narrative review to describe foundational principles and best practice activities. *Health Expectations* 2019;22(3):307-16.

44. Jackson T, Pinnock H, Liew SM, et al. Patient and public involvement in research: from tokenistic box ticking to valued team members. *BMC Med* 2020;18(1):79. doi: 10.1186/s12916-020-01544-7

45. Levelink M, Voigt-Barbarowicz M, Brütt AL. Priorities of patients, caregivers and health-care professionals for health research - A systematic review. *Health Expect* 2020;23(5):992-1006. doi: 10.1111/hex.13090

46. Bernges T, Iden L, Gielen R, et al. Forschen für uns! Welche Forschungsthemen interessieren von Depression Betroffene? *Psychiatr Prax* 2018;45(7):383-86. doi: 10.1055/s-0043-118147

47. Brütt AL, Bernges T, Magaard J, et al. Mitforschen, aber wie? Entwicklung und Evaluation eines Forschungstrainings fur Psychiatrieerfahrene. *Psychiatr Prax* 2017;44(2):99-104. doi: 10.1055/s-0041-108968

48. Brütt AL, Meister R, Bernges T, et al. Patient involvement in a systematic review: Development and pilot evaluation of a patient workshop. *Z Evid Fortbild Qual Gesundhwes* 2017;127-128:56-61. doi: 10.1016/j.zefq.2017.07.005

49. Levelink M, Eichstaedt HC, Meyer S, Brütt AL. Living with a left ventricular assist device: psychological burden and coping: protocol for a cross-sectional and longitudinal qualitative study. *BMJ open* 2020;10(10):e037017.

50. Seeralan T, Härter M, Koschnitzke C, et al. Patient involvement in developing a patient-targeted feedback intervention after depression screening in primary care within the randomized

For peer review only

controlled trial GET.FEEDBACK.GP. *Health Expect* 2021;24 Suppl 1:95-112. doi: 10.1111/hex.13039

51. Ernstmann N, Heuser C, Pfaff H. Health services research facilities at German universities. *Gesundheitswesen* 2018;82(4):313-17.
52. Reynolds J, Ogden M, Beresford R. Conceptualising and constructing 'diversity' through experiences of public and patient involvement in health research. *Res Involv Engagem* 2021;7(1):53. doi: 10.1186/s40900-021-00296-9
53. Thompson J, Bissell P, Cooper C, et al. Credibility and the 'professionalized' lay expert: Reflections on the dilemmas and opportunities of public involvement in health research. *Health* 2012;16(6):602-18.
54. Staley K, Elliott J, Stewart D, Wilson R. Who should I involve in my research and why? Patients, carers or the public? *Res Involv Engagem* 2021;7(1):41. doi: 10.1186/s40900-021-00282-1
55. Lander J, Langhof H, Dierks M-L. Involving patients and the public in medical and health care research studies: An exploratory survey on participant recruiting and representativeness from the perspective of study authors. *PloS one* 2019;14(1):e0204187.
56. Mills T, Lawton R, Sheard L. Advancing complexity science in healthcare research: the logic of logic models. *BMC medical research methodology* 2019;19:1-11.
57. Sammut R, Griscti O, Norman IJ. Strategies to improve response rates to web surveys: a literature review. *International Journal of Nursing Studies* 2021;123:104058.
58. Harris PA, Taylor R, Thielke R, et al. Research electronic data capture (REDCap)-A metadata-driven methodology and workflow process for providing translational research informatics support. *J Biomed Inform* 2009;42(2):377-81. doi: 10.1016/j.jbi.2008.08.010
59. IBM SPSS Statistics for Windows [program]. 29 version. Armonk, NY: IBM Corp., 2022.
60. Barbour RS. Doing focus groups. 2nd edition ed: SAGE 2018.
61. Guest G, Namey E, McKenna K. How Many Focus Groups Are Enough? Building an Evidence Base for Nonprobability Sample Sizes. *Field Methods* 2017;29(1):3-22. doi: 10.1177/1525822x16639015
62. Braun V, Clarke V. Using thematic analysis in psychology. *Qualitative research in psychology* 2006;3(2):77-101.
63. MAXQDA 2020 [program]. Berlin, Germany: VERBI Software, 2019.
64. Streiner DL. Health measurement scales a practical guide to their development and use. 5 ed ed: Oxford University Press 2015.
65. Mokkink LB, Terwee CB, Patrick DL, et al. The COSMIN study reached international consensus on taxonomy, terminology, and definitions of measurement properties for health-related patient-reported outcomes. *J Clin Epidemiol* 2010;63(7):737-45. doi: 10.1016/j.jclinepi.2010.02.006
66. Wild D, Grove A, Martin M, et al. Principles of Good Practice for the Translation and Cultural Adaptation Process for Patient-Reported Outcomes (PRO) Measures: report of the ISPOR Task Force for Translation and Cultural Adaptation. *Value Health* 2005;8(2):94-104. doi: 10.1111/j.1524-4733.2005.04054.x
67. Miller K, Chepp V, Willson S, Padilla J-L. Cognitive interviewing methodology: John Wiley & Sons 2014.

For peer review only

68. Polit DF, Beck CT, Owen SV. Is the CVI an acceptable indicator of content validity? Appraisal and recommendations. *Research in Nursing & Health* 2007;30(4):459-67. doi: https://doi.org/10.1002/nur.20199

69. Terwee CB, Bot SD, de Boer MR, et al. Quality criteria were proposed for measurement properties of health status questionnaires. *J Clin Epidemiol* 2007;60(1):34-42.

70. Frost MH, Reeve BB, Liepa AM, et al. What is sufficient evidence for the reliability and validity of patient-reported outcome measures? *Value in Health* 2007;10:S94-S105.

For peer review only

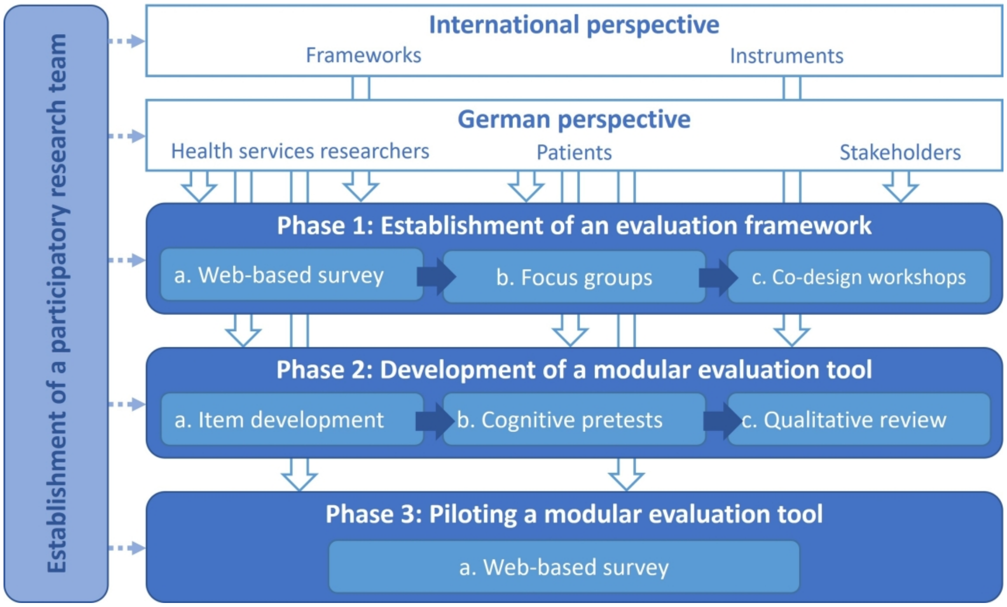

Figure 1: Overview of Study Design

226x137mm (300 x 300 DPI)
