# Supplementary material for: Development and piloting of a modular evaluation tool for patient and public involvement in health services research: protocol of a mixed-methods study
Source: BMJ Open. 2025 Dec 2;15(12):e110547. doi: 10.1136/bmjopen-2025-110547 (PMC12706111; doi:10.1136/bmjopen-2025-110547)
Supplement: Reviewer comments [file bmjopen-2025-110547.reviewer_comments.pdf]

PEER REVIEW HISTORY

BMJ Open publishes all reviews undertaken for accepted manuscripts. Reviewers are asked to complete a checklist review form and are provided with free text boxes to elaborate on their assessment. These free text comments are reproduced below.

ARTICLE DETAILS

Title (Provisional)

Development and Piloting of a Modular Evaluation Tool for Patient and Public Involvement in Health Services Research - Protocol of a Mixed-Methods Study

Authors

Seeralan, Tharanya; Oster, Lena; Jones, Julia; Mathie, Elspeth; Härter, Martin; Brütt, Anna Levke

---

This article was not externally reviewed at BMJ Open. Protocols that have been independently assessed prior to submission to BMJ Open are usually fast-tracked to publication on the grounds that further substantial changes will not be possible. This independent assessment will usually be external, independent review for both a substantial grant award from a non-commercial or government funder and ethics approval.
